# Development of a Human Preclinical Platform for the Identification of Neuroprotective Compounds

**DOI:** 10.1111/ejn.70328

**Published:** 2025-11-23

**Authors:** Raquel Guerrero González, Elif Nur Yilmaz, Stefanie Albrecht, Maurine Fucito, Damiana Pieragostino, Alina Schmidt, Simone König, Una FitzGerald, Tanja Kuhlmann

**Affiliations:** ^1^ Institute of Neuropathology University Hospital Münster Münster Germany; ^2^ Center for Advanced Studies and Technology G. d'Annunzio University of Chieti‐Pescara Chieti Italy; ^3^ Department of Innovative Technologies in Medicine and Dentistry University ‘G. d'Annunzio’ of Chieti‐Pescara Chieti Italy; ^4^ Core Unit Proteomics, Interdisciplinary Centre for Clinical Research, Medical Faculty University of Münster Münster Germany; ^5^ Science Foundation Ireland (SFI) Centre for Research in Medical Devices (CÚRAM) & Galway Neuroscience Centre University of Galway Galway Ireland

**Keywords:** axonal damage, demyelination, glutamate excitotoxicity, human iPSC, minocycline, multiple sclerosis, neuroprotection, oligodendrocyte differentiation, oxidative stress, pioglitazone

## Abstract

Multiple sclerosis (MS) is the most common inflammatory and demyelinating disease affecting the central nervous system (CNS). While immune‐modulating drugs can prevent new lesions by targeting lymphocyte activity, treating relapse‐independent disease progression remains challenging. Persisting CNS inflammation, leading to axonal and neuronal injury along with failure of compensatory mechanisms, such as brain plasticity and remyelination, drives disease progression. Thus, identifying neuroprotective and/or remyelination‐promoting compounds is urgently needed.

We developed an in vitro platform utilizing human‐induced pluripotent stem cell (iPSC)‐derived neurons and oligodendrocytes to assess neuroprotective and potentially promyelinating effects of selected compounds. We established assays mimicking MS pathophysiologies, such as neuronal loss and axonal injury. Proteomic analysis revealed modulation of molecular mechanisms. Findings were validated in an acute cuprizone (CPZ) mouse model.

We demonstrated that pioglitazone and minocycline protected against glutamate‐induced axonal injury, rotenone‐induced neuronal death and promoted oligodendrocyte differentiation. Proteomic analyses suggest that pioglitazone's neuroprotective effect may involve reducing mitochondrial reactive oxygen species (ROS) production via PGC‐1α and stabilizing axonal transport through GSK3β phosphorylation. Minocycline mainly impacted glutathione metabolism. In the cuprizone model, both compounds displayed neuroprotective effects but did not reduce demyelination or oligodendroglial loss. In summary, our findings demonstrate that human preclinical IPSC platforms can be used to characterize the neuroprotective properties of compounds and thus may aid the selection of drugs for clinical trials. Moreover, the platform's flexibility allows for the easy incorporation of additional disease‐specific phenotypic assays.

## Graphical abstract

Neuroprotection screening on neurons and OLs in oxidative stress and axonal damage assays. Analysis of neurite outgrowth, apoptosis, ROS, OL differentiation and proteomics. Cuprizone model assessed effects on neurodegeneration, inflammation and pro‐myelination.

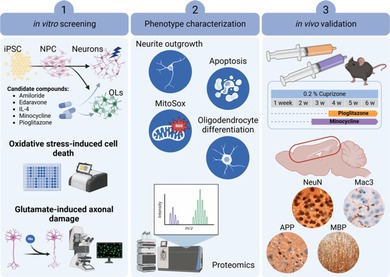

**Abbreviations**
AA: ascorbic acid
ALS: amyotrophic lateral sclerosis
APP: amyloid precursor protein
ASIC1: acid‐sensing ion channel 1
ATP: adenosine triphosphate
BBB: blood–brain barrier
BDNF: brain‐derived neurotrophic factor
CDKN1B: cyclin‐dependent kinase inhibitor 1B
CNS: central nervous system
CPZ: cuprizone
CSF: cerebrospinal fluid
DIV: days in vitro
DMEM: Dulbecco's modified eagle medium
DMSO: dimethyl sulfoxide
EAE: experimental autoimmune encephalomyelitis
EBs: embryoid bodies
FACS: fluorescence‐activated cell sorting
FASP: filter‐aided sample preparation
FBS: fetal bovine serum
GABA: gamma‐aminobutyric acid
GAP‐43: growth‐associated protein 43
GDM: glial differentiation medium
GDNF: glial‐derived neurotrophic factor
GIM: glial induction medium
Glu: L‐glutamic acid
GSK3β: glycogen synthase kinase 3 beta
GW: GW9662
hES: human embryonic stem
hiOL: human‐induced oligodendrocytes
IGF‐1: insulin‐like growth factor 1
IL‐4: interleukin 4
iPSC: induced pluripotent stem cell
KEGG: Kyoto Encyclopedia of Genes and Genomes
LAP3: leucine aminopeptidase 3
LC–MS/MS: liquid chromatography–tandem mass spectrometry
LFB: luxol fast blue
MBP: myelin basic protein
MEFs: mouse embryonic fibroblasts
MRI: magnetic resonance imaging
MS: multiple sclerosis
MTTP: microsomal triglyceride transfer protein
NEAA: non‐essential amino acids
NeuN: neuronal nuclear protein
NMDAR: *N*‐methyl‐d‐aspartate receptor
NPC: neural progenitor cells
NRF‐1/2: nuclear respiratory factor 1 and 2
NRF‐2: nuclear factor erythroid 2‐related factor 2
OLIG2: oligodendrocyte lineage transcription factor 2
OPC: oligodendrocyte progenitor cells
PAS: periodic acid Schiff
PBMC: peripheral blood mononuclear cell
PBS: phosphate‐buffered saline
PGC‐1α: peroxisome proliferator‐activated receptor gamma coactivator 1‐alpha
PI3K: phosphoinositide 3‐kinase
PMA: purmorphamine
PPARγ: peroxisome proliferator‐activated receptor gamma
ROS: reactive oxygen species
SAG: recombinant sonic hedgehog protein
SB: SB‐431542
T007: T0070907
TGF‐β3: transforming growth factor beta 3th
TH: Tyrosine hydroxylase
TUBB3: tubulin beta 3
VGLUT1: vesicular glutamate transporter 1

## 1 Introduction

Multiple sclerosis is the most frequent inflammatory and demyelinating disease in the central nervous system (CNS). Worldwide, it affects approximately 2.8 million people. The disease course is highly variable, and the accumulation of disability in MS is driven by a combination of relapse‐driven disease activity and relapse‐independent disease progression (Lublin et al. 2014; Tur et al. 2022). Relapse activity is primarily driven by immune cells that become activated in the periphery and then enter the CNS, resulting in the formation of focal inflammatory and demyelinating lesions. Whereas the formation of new focal lesions can be relatively successfully prevented by immune‐modulating drugs primarily targeting lymphocyte activation, proliferation or transmigration across the BBB, as shown by reduced relapse and MRI activity, the treatment of relapse‐independent disease progression remains challenging. The pathophysiology of disease progression is still poorly understood, but current concepts suggest that persisting CNS inflammation resulting in axonal and neuronal injury in combination with failure of compensatory mechanisms, such as brain plasticity and remyelination, drives disease progression (Kuhlmann et al. 2023). Therefore, the identification of neuroprotective and/or remyelination‐promoting compounds is an urgent but so far unmet need in MS.

Neurodegeneration in MS includes multiple mechanisms, which are non‐exclusive and may act in combination. In people with MS, the number of neurons as well as synapses is significantly reduced compared to nonneurological controls, both in grey matter lesions and in the normal appearing grey matter (Wegner et al. 2006; Jurgens et al. 2016; Carassiti et al. 2018). Inflammation in MS lesions correlates with acute axonal damage as well as axonal mitochondrial loss and dysfunction (Kuhlmann et al. 2002; Campbell and Mahad 2018). However, demyelination also contributes to axonal degeneration in MS. The myelin sheath not only ensures the saltatory conduction of action potentials but also protects against inflammatory attacks and provides trophic support to axons (Franklin et al. 2012). Additionally, loss of the myelin sheaths increases the energy demand of axons, which can only be insufficiently compensated by increased mitochondrial influx, promoting axonal degeneration (Licht‐Mayer et al. 2020; Licht‐Mayer et al. 2023). At the molecular level, oxidative stress and glutamate excitotoxicity have been proposed as important mediators of neuronal and axonal injury in MS (Correale et al. 2019; Woo et al. 2021). In MS, oxidative species are released from inflammatory cells, whereas excessive amounts of extracellular glutamate result from secretion by immune cells, release from dying cells and/or impaired glutamate uptake (Macrez et al. 2016; Birkner et al. 2020). High glutamate concentrations result in neuronal NMDAR activation and a sustained influx of calcium from the extracellular space (Hardingham and Bading 2010). Despite the increasing knowledge regarding the pathomechanisms driving axonal and neuronal injury, no neuroprotective treatment in MS has been approved and no positive phase 3 trials have been reported yet (Kaufmann et al. 2022; Chataway et al. 2024).

The aim of our study was to develop a human in vitro platform to test the neuroprotective and promyelinating properties of pharmacological compounds using human iPSC‐derived neurons and oligodendrocytes. Our in vitro platform includes assays to examine the effect of the compounds on axonal and neuronal injury as well as oligodendroglial cell death and differentiation. As a proof of concept study, we tested among other compounds pioglitazone, a peroxisome proliferator‐activated receptor gamma (PPARγ) agonist, the antibiotic minocycline and the cytokine IL4. In addition to their known mode of action, all three compounds have previously been shown to exhibit neuroprotective effects in vitro and/or in vivo in animal studies. In our study, all three compounds reduced axonal injury and neuronal loss in our human in vitro assays and had beneficial effects on oligodendroglial differentiation and survival. Proteomic analyses revealed molecular pathways induced by glutamate excitotoxicity in human iPSC‐derived neurons and identified shared and unique neuroprotective pathways activated by the different compounds. Pioglitazone reduced the loss of NeuN signal in the cortex and acute axonal injury in the corpus callosum in the cuprizone (CPZ) model, whereas minocycline only protected against the loss of NeuN signal. However, none of the compounds had a detectable effect on oligodendroglial cell death or demyelination in vivo.

## 2 Methods

### 2.1 Generation of Neural Progenitor Cells (NPCs)

NPCs were generated from induced pluripotent stem cells (iPSCs) using a previously described method involving the application of small molecules (Reinhardt et al. 2013). In brief, iPSC colonies from Passages 10–15 were mechanically dissected and detached from mouse embryonic fibroblasts (MEFs) using enzymatic treatment. Fragments of iPSC colonies were collected by sedimentation, suspended in human embryonic stem (hES) cell medium, consisting of DMEM‐F12 (Invitrogen) with 20% Knockout serum replacement (Gibco), 1% penicillin/streptavidin (Sigma‐Aldrich), 1% L‐glutamine (Sigma‐Aldrich), 1% NEAA (Sigma‐Aldrich) and 0.2% 2‐mercaptoethanol (Thermo Fisher Scientific) and supplemented with specific small molecules: 10 μM SB‐431542 (Ascent Scientific), 1‐μM dorsomorphin (Tocris), 3 μM CHIR99021 (CHIR; Axon Medchem) and 0.5‐μM purmorphamine (PMA; Alexis). The cell fragments were then cultured as embryoid bodies (EBs) in petri dishes. After 2 days, the medium was replaced with N2B27 medium, a combination of equal parts DMEM‐F12 and Neurobasal (Invitrogen), supplemented with 1:200 N2 supplement (Invitrogen), 1:100 B27 supplement without vitamin A (Invitrogen), 1% penicillin/streptomycin/glutamine, and the same small molecule supplements as previously mentioned. On Day 4, SB‐431542 and dorsomorphin were removed, and 150‐μM ascorbic acid (AA; Sigma‐Aldrich) was introduced to the medium. On Day 6, the EBs were dissociated into smaller fragments and plated on 12‐well plates (Nunc) coated with Matrigel (Matrigel, growth factor reduced, high concentration; BD Biosciences). The plates were supplied with NPC expansion medium, which consisted of N2B27 medium supplemented with CHIR, PMA and AA. The cells were subsequently passaged at ratios ranging from 1:10 to 1:15 every five to 6 days.

### 2.2 Generation of Human iPSC‐Derived Neurons

Neurons were generated from NPCs according to the procedure described by Reinhardt and colleagues (Reinhardt et al. 2013). In brief, NPCs from passages 10 to 20 were isolated and plated onto Matrigel‐coated plates at a density of 1.25 × 10^5^ cells per well (−1 days of in vitro differentiation (−1 DIV)), using NPC expansion medium. One day after plating (0 DIV), the medium was switched to neuronal induction medium (N2B27 supplemented with 1‐μM SAG, 100‐μM AA, 2‐ng/mL BDNF, 2‐ng/mL GDNF). At 6 DIV, the neuronal induction medium was replaced with neuronal maturation medium (N2B27 supplemented with 2‐ng/mL BDNF, 2‐ng/mL GDNF, 1‐ng/mL TGF‐β3 (all from PeproTech), 100‐μM AA and 100‐μM dbcAMP) (Sigma‐Aldrich), with 5‐ng/mL Activin A additionally only for 4 days (6 DIV to 10 DIV). To prevent overcrowding, neuronal cultures were split at 10 DIV and plated in the final format suitable for each assay. The medium was refreshed every other day, and the cultures were typically analysed after 23 days in maturation medium, unless otherwise specified.

### 2.3 Viability and Oxidative Stress Assay

Neurons were replated at 10 DIV into Matrigel‐coated 96‐well plates at a density of 1 × 10^4^ cells per well. At 21 DIV cells were treated with the following compounds: Amiloride, Edaravone, Minocycline or Pioglitazone (all from Sigma‐Aldrich) at 1 μM (in DMSO) or IL‐4 (PeproTech) at 50 ng/mL (in DMSO) or DMSO (0.1%) as a vehicle for 24 h, followed by treatment with the stress agent at 22 DIV for an additional 24 h (Rotenone (Sigma‐Aldrich) 500 nM or Taxol (Sigma‐Aldrich) 100 nM). For the assays with the PPARγ antagonists, the cells were treated at 21 DIV with pioglitazone (1 μM) alone and in combination with GW9662 (10 μM), T0070907 (10 μM) or vehicle (DMSO 0.1%) for 24 h, followed by 6 h of rotenone treatment at 22 DIV. To test GSK3β blockers hiNs at 21 DIV were treated with pioglitazone (1 μM), CHIR (10 μM), LiCl (5 mM), tideglusib (2.5 μM) or vehicle (DMSO 0.1%) for 24 h, followed by 6 h of rotenone treatment at 22 DIV. Subsequently, cell viability was assessed using the CellTiter Glo Assay, Luminescence (Promega) on 23 DIV.

### 2.4 Glutamate‐Induced Axonal Damage Assay

For glutamate‐induced axonal damage, neurons were replated at 10 DIV into Matrigel‐coated 48‐well plates at a density of 7.5 × 10^4^ cells per well. At 21 DIV cells were treated with the compounds (Amiloride, Edaravone, Minocycline or Pioglitazone at 1 μM or IL‐4 at 50 ng/mL) or vehicle (DMSO 0.1%) for 24 h, followed by treatment with L‐glutamic acid (Sigma‐Aldrich, 1‐mM diluted in HCl 1 M) or the same volume of HCl 1 M as negative control. For the assays with PPARγ antagonists, cells were treated at 21 DIV with pioglitazone (1 μM) alone and in combination with GW9662 (10 μM) or T0070907 (10 μM) for 24 h. For GSK3β blocker assays, pioglitazone (1 μM), CHIR (10 μM), LiCl (5 mM), tideglusib (2.5 μM) or vehicle (DMSO 0.1%) were administered at 21 DIV for 24 h, followed by 24 h of glutamate exposure (1 mM) or HCl as control. On 23 DIV, the cells were fixed for immunocytochemical staining with anti‐SMI31 and imaged with a confocal microscope.

### 2.5 Neurite Length Assay

At 10 DIV neurons were replated into a Matrigel‐coated 24‐well plate at a density of 5 × 10^4^ cells per well in Neuronal Differentiation medium plus compounds (Minocycline or Pioglitazone at 1 μM or IL‐4 at 50 ng/mL) or vehicle (DMSO 0.1% vol/vol) for 24 h. After 24 h of incubation, the cells were fixed for immunocytochemical staining with anti‐tubulin β3 (TUBB3) and imaged with a confocal microscope.

### 2.6 Cleaved Caspase‐3

For the cleaved caspase‐3 assay, neurons were replated at 10 DIV into Matrigel‐coated 24‐well plates at a density of 1.5 × 10^5^ cells per well. At 21 DIV cells were treated with the compounds (Minocycline or Pioglitazone at 1 μM or IL‐4 at 50 ng/mL) or vehicle (DMSO 0.1% vol/vol) for 24 h, followed by treatment with rotenone 100 nM for 6 h. On 23 DIV the cells were fixed for immunocytochemical staining with anti‐cleaved caspase‐3 and anti‐TUBB3 and imaged with a confocal microscope.

### 2.7 MitoSOX

For the MitoSOX assay to measure mitochondrial reactive oxygen species (ROS), neurons were replated at 10 DIV into 12‐well plates at a density of 3.0 × 10^5^ cells per well. At 21 DIV, cells were treated with the compounds (Minocycline or Pioglitazone at 1 μM or IL‐4 at 50 ng/mL) or DMSO 0.1% vol/vol as a vehicle for 24 h, followed by treatment with rotenone 100 nM for 24 h. On 23 DIV, cells were washed with 1× HBSS buffer once. Then, 2.5 μM of MitoSOX Red reagent (Invitrogen) diluted in 1× HBSS was added to all conditions except the negative control and incubated at 37°C for 15 min. After incubation, all wells were washed once more with 1× HBSS, and the cells were prepared for flow cytometry.

Neurons were detached with Accutase (5 min at 37°C), and the reaction was stopped using split solution (1% BSA fraction V in DMEM low glucose). The suspension was centrifuged at 250 ×*g* for 5 min, to be then resuspended in 500‐μL FACS buffer (0.5% BSA in PBS) and triturated into a single‐cell suspension before FACS analysis using a 35 μM strainer (Cat. No. 10585801, Fisher Scientific). For readout, MitoSOX Red was excited using the blue laser at 488 nm wavelength and detected at PE emission optimum (576 nm) in fluorescence cytometry.

### 2.8 Immunocytochemistry (ICC)

For confocal or fluorescence microscopy, cells were cultured on Matrigel‐coated glass coverslips in 24‐well plates. Neurons were fixed with 4% paraformaldehyde in PBS (Invitrogen) for 15 min at room temperature (RT) and washed three times with PBS. Blocking and permeabilization were performed using 0.1% Triton X‐100 (Sigma‐Aldrich) in PBS, 5% fetal bovine serum (FBS) and 5% normal goat serum (NGS, or 10% FBS for staining of Sox1) in PBS for 45–60 min at RT. Subsequently, coverslips were washed three times with PBS, and primary antibodies (Table S1) were applied overnight at 4°C in 5% FBS/NGS. The next day, the cells were washed three times with PBS (5 min each) and then incubated with the secondary antibodies (1 h, RT) (Table S1).

For fluorescence microscopy, a DAPI counterstaining for nuclei was performed prior to the last three PBS washes, and the cells were then imaged in a fluorescence microscope CKX53 (Olympus). For confocal microscopy, cells were washed three times in PBS after incubation with the secondary antibody, mounted with Roti‐Mount FluorCare DAPI medium (Roth) and imaged on a Zeiss LSM 700 confocal microscope.

### 2.9 iPSC‐Derived Oligodendroglial Differentiation Assay

The protocol starts with NPCs that have a stable integration of the SON‐cassette into the AAVS1 locus of their genome. The SON factors are controlled by a Tet‐on system, which induces their expression via the addition of a tetracyclic molecule, doxycycline, as described previously (Ghelman et al. 2021).

Approximately 6.5 × 10^4^ cells per well were plated into a Matrigel‐coated 12‐well plate with NPC medium. After 24 h, the medium was changed to NPC medium containing 1‐μg/mL doxycycline; 48 h after the doxycycline addition, the medium was changed to glial induction medium (GIM). GIM contains 1% B27 supplement without vitamin A, 0.5% N2 supplement, 1% Pen/Strep, 2‐mM L‐glutamine, 1‐μM SAG, 200‐μM L‐ascorbic acid, 10‐ng/mL T3, 10‐ng/mL IGF‐1, 10‐ng/μL PDGF‐AA, 10‐ng/μL human NT3 and 1:1000 Trace element B diluted in DMEM/F12. This time point of the experiment is defined as Day 0 of differentiation (DIV 0). On DIV 2, the medium was switched to glial differentiation medium (GDM) containing 1% B27 supplement without vitamin A, 0.5% N2 supplement, 1% Pen/Strep, 2‐mM L‐glutamine, 200‐μM L‐ascorbic acid, 60‐ng/mL T3, 10‐ng/mL IGF‐1, 10‐ng/μL human NT3, 200‐μM dbcAMP and 1:1000 Trace element B diluted in DMEM/F12. On DIV 7, cells were detached by Accutase treatment for 10 min at 37°C. The activity of Accutase was inactivated by adding split solution (in a dilution of 1:10). Then, the cell suspension was centrifuged for 5 min at 200 rcf, and the supernatant was discarded. Subsequently, the pellet was resuspended in GDM. The cells were counted using a Neubauer chamber and plated in the desired number according to the performed experiment. The medium was changed 48 h after the seeding and every second day until the end of differentiation. On DIV 16, doxycycline was removed from the culture medium. Lastly, the differentiation was terminated on DIV 21.

### 2.10 Compound Screening for Oligodendrocyte Differentiation and Maturation

To observe the effects of the candidate compounds on oligodendrocyte differentiation, the full media conditions GIM and GDM were reduced to only essential/minimal ingredients. GIM was adapted into minimal condition‐GIM, lacking T3, while GDM was adapted into minimal condition‐GDM lacking NT3, T3, IGF‐1 and Trace B elements, as described earlier with slight modifications (Ehrlich et al. 2017). At DIV 7, cells were detached, triturated into a single‐cell suspension and reseeded at densities of 1.2 x 10^4^ cells onto PLL/laminin‐coated 48‐well plates, or 2.4 × 10^4^ cells onto PLL/laminin‐coated 24‐well plates. Cells were allowed to recover in minimum GDM for 48 h. At DIV 9, cells were treated with vehicle alone [0.1% (vol/vol) DMSO] as a negative control, 60 ng/mL T3 as a positive control, or with a 1‐μM candidate drug dissolved in DMSO in minimum GDM. The drug candidates include benztropine, clemastine, interleukin‐4, minocycline and pioglitazone. The medium was changed every other day, and doxycycline was removed from the medium on DIV 16. The protocol was terminated on differentiation DIV 21. For ICC, cells were fixed with 4% PFA and stained with anti‐O4 and anti‐MBP antibodies.

### 2.11 OPC Proliferation Assay

The proportion of proliferating OPCs in the culture was analysed via Ki67/O4 double staining. Cells were seeded and treated with 1‐μM hit compounds (IL‐4, minocycline and pioglitazone), and differentiation was terminated at DIV 14. Fixed cells were stained with an anti‐O4 antibody and subsequently with an anti‐Ki67 antibody.

### 2.12 OPC Oxidative Stress‐Induced Cell Death

The cleaved form of caspase‐3 is responsible for most proteolysis during apoptosis; therefore, detecting cleaved caspase‐3 is considered a reliable marker for cells dying or already dead by apoptosis (Crowley and Waterhouse 2016). To detect the anti‐apoptotic effect of candidate compounds, stress was induced with 1‐μM rotenone for 24 h at 18 DIV. Then, stressed oligodendrocytes were treated with the candidate drugs (minocycline and pioglitazone at 1 μM or IL‐4 at 50 ng/mL) for 48 h. At DIV 21, cells were fixed and then stained with cleaved caspase‐3 to detect apoptotic cells.

### 2.13 Sample Preparation and LC–MS/MS Acquisition for Proteomic Analysis

Cell pellets were chemically lysed in lysis buffer (6‐M urea, 10‐mM Tris HCl, 5‐mM DTT, 1% Triton X‐100, 2% CHAPS, pH 7.5). Subsequently, the samples were mechanically lysed by manual pipetting and sonication (Sonicator U200S control, IKA Labortechnik, 80% amplitude, 10 s, 3 cycles). Protein concentration was determined by Bradford assay (Bio‐Rad) using bovine serum albumin (Sigma‐Aldrich) to generate the standard curve. Samples were prepared according to manufacturer instructions, and absorbance was measured at 595 nm.

From each sample, 30 μg of protein was digested according to the filter‐aided sample preparation (FASP) protocol (Wisniewski et al. 2009). In short, the protein suspension was loaded into a 10‐kDa nanostep filter equipped with an Omega membrane (VWR international), and protein digestion was performed according to an adapted protocol (Distler et al. 2016). Briefly, the protein suspension was washed with urea buffer (8‐M urea, 100‐mM Tris, pH 8.5 in H2O). Then, it was reduced and alkylated with 100 μL of DTT (8‐mM DTT in urea buffer, Sigma‐Aldrich) and 100 μL of IAA (50 mM IAA in urea buffer, Sigma‐Aldrich), respectively. Finally, proteins were digested using trypsin (Promega) with an enzyme‐to‐protein ratio of 1:50 w/w in 50‐mM ammonium bicarbonate (J.T. Baker). The samples were incubated at 37°C overnight. Samples were eluted from the filter by centrifugation (15 min, 12,400 rpm, RT), and peptide samples were acidified with 10% FA (Fluka).

Each sample was analysed in triplicates. LC–MS/MS platform was composed of an UltiMate 3000 nanoflow‐ultra high‐performance LC system coupled to an Orbitrap Fusion Tribrid mass spectrometer via a nanoESI source (all from Thermo Fisher Scientific). Peptides were concentrated onto a PepMap C18 precolumn cartridge (5‐μm particle size, 5 mm length × 300‐μm internal diameter) before being separated on an EASY‐Spray PepMap rapid separation liquid chromatography C18 column (2‐μm particle size, 25‐cm length × 75‐μm internal diameter) using a two‐step linear gradient of 100 min (from 5% to 21% CAN (100% ACN, 0.1% Fad) in 90 min and then from 21% to 55% ACN in 10 min) followed by 25 min of wash and column equilibration. Precursor spectra (MS1) were acquired in positive mode at high resolution (240.000 at 200 m/z), and fragment spectra (MS2) were acquired for the 10 most abundant precursor ions (data dependent acquisition) using higher energy C‐trap dissociation (30% normalized collision energy). Quadrupole isolation with a 1,6 m/z window and dynamic exclusion were used. The system was set to record at specific automatic gain control targets and maximum injection time.

Protein data were processed using Proteome Discoverer (Thermo Fisher Scientific, version 2.4) with Sequest HT as the search engine, using the human protein database from UniProt. Specific settings included trypsin as the enzyme, carbamidomethylation of cysteines as a fixed modification, and oxidation of methionines as a variable modification. Peptide‐level filtering was achieved using the Percolator algorithm to maintain a false discovery rate below 0.01. Quantification was based on precursor abundance, and normalization was done according to total peptide amounts. Protein abundance ratios were calculated, with statistical significance determined via *t*‐tests (*p*‐value < 0.05). Following quantification, I analysed the relative protein expression between different experimental conditions using GraphPad Prism 8, identifying upregulated (ratio > 2) and downregulated (ratio < 0.5) proteins. Only those comparisons with *p*‐value < 0.05 were considered. Enrichment analysis of these proteins was performed using EnrichR (Chen et al. 2013; Kuleshov et al. 2016; Xie et al. 2021) with the KEGG Human 2021 database, and the top 10 pathways (top 10 lowest *p*‐values) were further analysed using STRING (version 11.5) to visualize interacting protein clusters within biological processes and cellular components. The following settings were applied: Full STRING network was selected as the network type, with edges representing evidence of interactions. All available active interaction sources were included, and the minimum required interaction score was set to 0.700 (high confidence). No limit was imposed on the maximum number of interactors to display.

### 2.14 CPZ Model

For the CPZ model all C57Bl/6 mice were obtained from Janvier Labs, France. Eight‐week‐old, male C57BL/6 mice were fed ad libitum with powdered chow containing 0.2% CPZ (Sigma‐Aldrich) for 6 or 10 weeks. Weight and health status were recorded daily. As a control, healthy C57BL/6 mice were sacrificed at 18 weeks.

For the first group, CPZ was fed for 6 weeks, and 15 mg/kg/day of pioglitazone was administered p.o. for the last 3 weeks of demyelination (Weeks 4 to 6), or the equivalent volume of vehicle solution (0.5% Methocel A4M). For the second group, again with 6 weeks of CPZ, 25 mg/kg/day of minocycline was applied i.p. for the last 4 weeks of demyelination (Weeks 3 to 6), or the equivalent volume of vehicle solution (0.9% NaCl). Mice were sacrificed by transcardial perfusion. Brain hemispheres were removed and fixed in 4% PFA for histochemical analysis.

### 2.15 Immunohistochemistry (IHC)

After fixation, brain hemispheres were embedded in paraffin and subsequently sectioned (4 μm). For IHC, a biotin‐streptavidin peroxidase technique (K5001, Dako) or fluorescence staining (anti‐rabbit Cy3, anti‐mouse 488 antibodies) and an automated immunostainer (AutostainerLink 48, Dako) were used. Sections were pretreated with citrate buffer (pH 6), Target Retrieval Solution (pH 6.1; Dako) or TE buffer (pH 9). Primary antibodies are listed in Table S1.

Luxol fast blue (LFB) was used to detect phospholipids or lipoproteins in myelin, and periodic acid Schiff (PAS) was used to detect glycoproteins (LFB‐PAS). Slides were incubated with alum haematoxylin for counterstaining nuclei.

### 2.16 Quantifications

For the in vitro experiments, at least three independent experiments were performed with three or four technical replicates (i.e., wells) for each experiment.

For the histological analyses slides were blindly analysed in an Olympus BX51 using an ocular morphometric grid or after digitalisation with Ocus40 (Grundium Finland; 20× objective magnification). Quantification of NeuN+ neurons, MAC3+ macrophages/microglia, APP+ spheroids, Olig2+ oligodendroglial cells, NOGOA+ mature oligodendrocytes, CD3+ T‐cells in the cortex and/or the corpus callosum was performed manually, based on at least 12 random fields in the corpus callosum and 30 random fields in the cortex per animal. The resulting average cell number was divided by the field area, obtaining cells per mm^2^. The extent of demyelination in the cortex was examined by MBP IHC and quantified employing ImageJ Fiji software, as previously described and applied (Crowe and Yue 2019; Yilmaz et al. 2023). NeuN+ neurons were determined by counting positive cell bodies. The extent of demyelination in the caudal corpus callosum was determined in a blinded manner in sections stained for LFB‐PAS. A semi‐quantitative score from myelin was applied as follows: 0 = no myelin, 1 = 1%–33% myelination, 2 = 34%–67% myelination and 3 = more than 68% myelination in the analysed area (Preisner et al. 2015).

### 2.17 Statistics

Statistical analyses were conducted using GraphPad Prism 8 (GraphPad Software Inc.), with data presented as mean ± standard error of the mean (SEM). For proteomic data, individual comparisons were assessed using a two‐tailed Student's t‐test. Experimental results were analysed using one‐way ANOVA, followed by Dunnett's or Tukey's multiple comparison tests. For LFB‐PAS semiquantification, a Kruskal–Wallis test with Dunn's multiple comparison was performed. Statistical significance was determined with *p*‐values less than 0.05 (*p < 0.05, **p < 0.01, ***p < 0.001 and ****p < 0.0001).

## 3 Results

### 3.1 Generation of Human Glutamatergic Neurons

**FIGURE 1:**
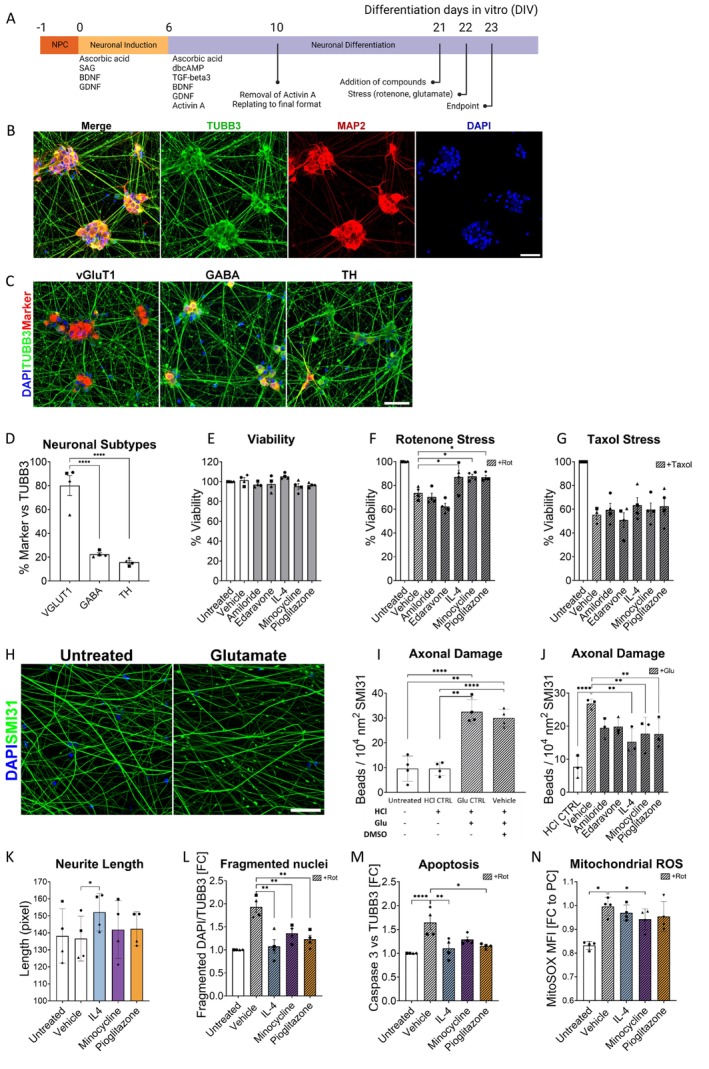
Compound screening with human iPSC‐derived neurons. (A) Timeline of the neuronal differentiation through the application of small molecules in the neuronal induction medium (0 to 6 DIV), and the neuronal differentiation medium (6 to 23 DIV). Compounds were applied at 21 DIV for 24 h and subsequently stressed with rotenone, glutamate or taxol for additional 24 h. (B) Immunocytochemistry for the neuronal markers TUBB3 and MAP 2 show ganglia‐like distribution of the neuronal population in culture. Scale bar: 50 μm. (C–D) Immunocytochemistry for neuronal markers showed that the neuronal population at 23 DIV was composed of mainly glutamatergic neurons (VGLUT1+), followed by GABAergic (GABA+) and dopaminergic (TH+) in a lesser amount. Scale bar: 50 μm. (E) CellTiterGlo assay to assess viability showed no toxic effect by the candidate compounds. (F) Incubation with rotenone led to a reduction to 50% viability. Pretreatment with compounds can partially rescued cell survival. (G) Incubation with taxol similarly reduced viability to approx. 50%. However, pretreatment with the compounds could not rescue cell survival. (H) Treatment of iPSC‐derived neurons with glutamate 1 mM for 24 h lead to neurofilament‐positive axonal beads (SMI31). (I) Quantification of axonal beads against neurofilament‐positive area (SMI31+) corroborated the significant difference in the number of beads after glutamate treatment. (J) Quantification of axonal beads after pretreatment with the compounds. IL‐4, minocycline and pioglitazone significantly reduced the number of spheroids. (K) Quantification of the neurite length assay after 24 h of incubation with the compounds after replating. Only IL‐4 significantly increased neurite length. 0.1% DMSO served as vehicle. (L) All three compounds reduce the number of fragmented nuclei after rotenone treatment (M) IL‐4 and pioglitazone significantly reduced the number of cleaved caspase‐3‐positive neurons after rotenone stress. (N) Minocycline significantly reduced mitochondrial ROS content after rotenone stress as measured in a MitoSOX assay. *n* = 4 independent experiments. *p ≤ 0.05, **p ≤ 0.01, ***p ≤ 0.001 and ****p ≤ 0.0001; one‐way ANOVA followed by Dunnett's multiple comparison test.

iPSCs were differentiated into NPCs by in vitro patterning using small molecules (Figure 1A). These NPCs are an intermediate cell type between iPSCs and terminally differentiated CNS cells and can be differentiated into neurons, astrocytes and oligodendrocytes (Reinhardt et al. 2013). NPC identity was validated by ICC for NESTIN and the transcription factor SOX1 (Figure S1). NPCs were differentiated into neurons after 6 days of prepatterning and 17 days of maturation; the percentage of neurons at 23 DIV was 79.60% ± 2.98, as assessed by ICC for TUBB3 (Figure 1B). To characterize the neuronal subtypes, we quantified glutamatergic (VGLUT1+), GABAergic (GABA+) and dopaminergic (TH+) neurons in relation to TUBB3+ neurons at 23 DIV. Glutamatergic neurons constitute the majority (80.1% ± 8.4%), followed by GABAergic (22.5% ± 1.3%) and dopaminergic (15.8% ± 1.4%) neurons (Figure 1C,D).

### 3.2 IL‐4, Minocycline and Pioglitazone Reduced Glutamate‐Induced Axonal Injury and Oxidative Stress‐Mediated Neuronal Cell Death

In order to facilitate a possible clinical translation, we focused on drugs that are already in clinical use and have shown neuroprotective effects in rodent in vitro or in vivo studies. Amiloride is a diuretic and inhibitor of acid‐sensing ion channel 1 (ASIC1) blocking the influx of Na^+^ and Ca^2+^, whereas Edaravone is a free radical scavenger used as a treatment for ALS. Minocycline is a tetracycline antibiotic also known for its anti‐inflammatory properties, while the PPARγ agonist pioglitazone is an anti‐diabetic medication, which also promotes mitochondrial functions. All four compounds demonstrated neuroprotective effects either in rodent in vitro experiments or in animal models of MS, ischemic or traumatic brain injury (Wilkins et al. 2004; Maier et al. 2007; Vergo et al. 2011; Wang et al. 2011; Licht‐Mayer et al. 2023). Moreover, we also included the cytokine IL‐4, which has been shown to induce neurite outgrowth but also to induce an anti‐inflammatory phenotype in myeloid cells (Francos‐Quijorna et al. 2016; Vogelaar et al. 2018).

First, we tested the potential cytotoxic effects of the compounds by measuring intracellular ATP levels, which correlate with the number of viable cells. We observed no significant adverse effects of the compounds on cellular viability (Figure 1E). Since oxidative stress has been shown to mediate axonal injury in MS (Gilgun‐Sherki et al. 2004; Ohl et al. 2016), we investigated the survival of neurons after the induction of oxidative stress induced by the mitochondrial chain I inhibitor rotenone. The application of rotenone significantly decreased cell survival after 24 h to 73.65% ± 5.31%, as determined by ATP levels. Pretreatment with IL‐4, minocycline, and pioglitazone for 24 h resulted in a mild but significant increase in survival compared to vehicle (Figure 1F).

To determine whether the protective effect was specific to oxidative stress, we exchanged rotenone for the microtubule stabilizer taxol, which induces cell death without affecting the activity of the respiratory chain (Gornstein and Schwarz 2014). Taxol reduced the viability to a similar extent as rotenone (83.61% ± 5.69%); however, none of the compounds was able to reduce taxol‐induced cell death (Figure 1G). Since it is widely accepted that accumulating axonal loss contributes to disease progression in MS (Reynolds et al. 2011), we established an in vitro model of axonal damage. The application of glutamate (1 mM for 24 h) led to the formation of axonal beads along the axons of iPSC‐derived neurons (Figure 1H–I), as reported by others in primary rat cortical neurons (Chung et al. 2005), without impairing cell viability (Figure S2A). These swellings can be identified by staining for the unphosphorylated neurofilament‐H marker SMI31, and they precede axonal transection (Takeuchi et al. 2005). Pretreatment of iPSC‐derived neurons for 24 h with either IL‐4, minocycline or pioglitazone, but not amiloride or edaravone, significantly reduced the number of axonal beads in relation to the neurofilament‐positive area (SMI‐31+, Figure 1J).

In contrast to amiloride and edaravone, IL‐4, minocycline and pioglitazone reduced glutamate‐induced axonal injury and oxidative stress‐mediated neuronal cell death; therefore, we decided to focus on IL‐4, minocycline and pioglitazone in the subsequent experiments.

We performed a neurite outgrowth assay to quantify the effects of the compounds on neurite length (Figure S2B). At 10 DIV, cells were treated for 24 h with the different compounds. Only IL‐4 caused a mild but significant increase in neurite length, when compared to the vehicle control (Figure 1K).

To further analyse the effects of the three remaining compounds on apoptotic cell death, we quantified the number of fragmented nuclei and cleaved caspase‐3‐positive neurons (Caspase‐3+, TUBB3+) after rotenone treatment (100 nM, 6 h) (Figure S2C–D). All three compounds reduce the number of fragmented nuclei. All three compounds also showed a trend to reduce the number of activated caspase‐3+ neurons; however, only following IL‐4 and pioglitazone treatment did the effect reach significance compared to vehicle (Figure 1L–M). Lastly, because increased ROS and mitochondrial dysfunction are believed to contribute to disease progression in MS (Campbell and Mahad 2018), we determined whether the candidate compounds reduce ROS production. As expected, rotenone exposure (100 nM, 24 h) increased mitochondrial ROS production in neurons, as determined by the mitochondrial superoxide indicator MitoSOX (Figure 1N). Following pretreatment with the three candidate compounds, only minocycline significantly reduced MitoSOX intensity compared to the DMSO control (Figure 1N).

In summary, our results demonstrate that IL4, pioglitazone and minocycline protected human iPSC‐derived neurons against glutamate‐induced axonal damage. Furthermore, all three compounds reduced rotenone‐induced oxidative cell death, evidenced by viability and/or caspase‐3 assays. Additionally, minocycline mildly reduced ROS production in neurons.

### 3.3 Identification of Pathways Affected by IL‐4, Pioglitazone and Minocycline in Neurons

To dissect the pathways by which the compounds prevent acute axonal damage, we performed LC–MS/MS. Treatment of human iPSC‐derived neurons with glutamate resulted in the upregulation of 128 proteins and the downregulation of nine proteins compared to untreated control. In contrast, iPSC‐derived neurons cultured in the presence of glutamate and treated with pioglitazone resulted in the downregulation of 235 and the upregulation of four proteins compared to neurons exposed to glutamate alone, whereas treatment with IL‐4 upregulated the majority of differentially expressed proteins (202 upregulated and two downregulated). Minocycline had only mild effects on the proteome (12 upregulated and nine downregulated proteins) (Figure S3 and Table S2).

**FIGURE 2:**
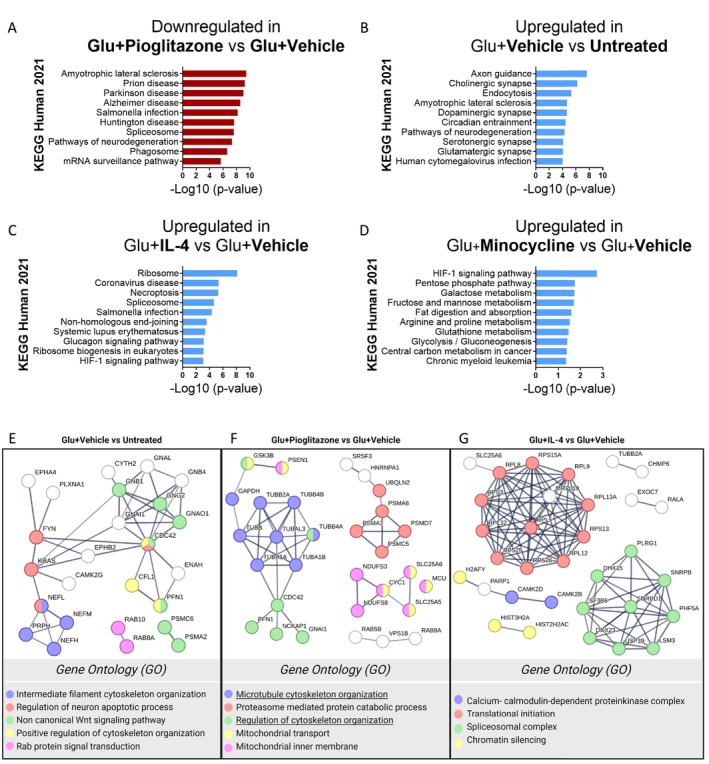
Proteomic analysis of glutamate‐induced axonal damage assay. Pathway enrichment of up‐ (blue) or downregulated (red) proteins in (A) glutamate‐stressed neurons (Glu + Vehicle) compared untreated neurons (Untreated), and (B) Glu + Pioglitazone, (C) Glu + IL‐4 and (D) Glu + Minocycline compared to Glu + Vehicle compared. Pathways are based on KEGG Human 2021 database. *p*‐Values < 0.05 were considered statistically significant, and the 10 lowest *p*‐values were depicted in the graph. A list of the proteins involved in each pathway can be found in Table S3. (E–G) The proteins involved in the top 5 KEGG pathways were analysed in STRING, and clusters were highlighted according to the GO terms. Line thickness indicates the strength of data support in the STRING network. Proteins depicted in white do not belong to any of the selected GO terms.

Pathway enrichment analyses using Enrich R and KEGG Human 2021 database demonstrated that glutamate treatment resulted in the upregulation of proteins belonging to categories such as axon guidance, cholinergic synapse, endocytosis, amyotrophic lateral sclerosis and pathways of neurodegeneration (Figure 2A). In contrast, treatment with pioglitazone reduced the expression of proteins belonging to KEGG categories, such as amyotrophic lateral sclerosis or pathways of neurodegeneration (Figure 2B). IL‐4 treatment was associated with the upregulation of proteins associated with KEGG terms, such as ribosome, spliceosome and necroptosis (Figure 2C), whereas minocycline treatment resulted in the upregulation of selected proteins involved in metabolic pathways (pentose phosphate pathway, galactose, fructose and mannose metabolism, fat digestion and absorption and glycolysis/gluconeogenesis) (Figure 2D and Table S3).

To dissect protein interactions and clusters, proteins belonging to the top five enriched pathways were analysed further using STRING. Glutamate induced the upregulation of proteins involved in cytoskeleton organization, e.g., neurofilament subunits (NEF‐H, NEF‐M and NEF‐L), and proteins involved in the remodelling of the cytoskeleton (FYN, CDC42 and guanine‐binding protein), in line with the observation of axonal swellings after glutamate exposure (Figure 2E). In contrast, treatment with pioglitazone resulted in the downregulation of proteins related to the GO terms ‘microtubule cytoskeleton organisation’ and ‘regulation of cytoskeleton organisation’ (including GSK3β), as well as ‘proteasome‐mediated ubiquitin‐dependent protein catabolic process’, and ‘mitochondrial inner membrane’ (Figure 2F). Treatment with IL‐4 resulted in the upregulation of proteins belonging to the GO terms ‘translational initiation’ and ‘spliceosomal complex’ clusters (Figure 2G). Of importance also are proteins involved in ‘chromatin silencing’ and ‘calcium‐ and calmodulin‐dependent protein kinase complex’ (CAMK2D, CAMK2B), two isoforms of the CAMKII family, which play critical roles in regulating calcium homeostasis and actin cytoskeleton reorganization, particularly in the context of synaptic plasticity (Okamoto et al. 2007; Lisman et al. 2012).

Treatment with minocycline induced the upregulation of only a few selected proteins, like LAP3, PFKL, CDKN1B and MTTP. LAP3 is involved in glutathione biosynthesis and glutamate catabolic processes (Cappiello et al. 2004). PFKL and MTTP are involved in metabolic pathways (pentose phosphate pathway, galactose metabolism, fructose and mannose metabolism, fat digestion and absorption and glycolysis/gluconeogenesis). CDKN1B is a cyclin‐dependent kinase inhibitor that, in the cytoplasm, associates with microtubules and it is required for proper axonal transport, neuronal migration and dendritic spine formation (Garcia‐Osta et al. 2022). Importantly, CDKN1B was downregulated in glutamate‐treated neurons (Table S2).

In summary, our proteome analyses demonstrate that exposure of human iPSC‐derived neurons to glutamate results in the upregulation of proteins involved in the remodelling of the cytoskeleton. Although treatment with pioglitazone, IL‐4 and minocycline all reduce the extent of acute axonal damage, the proteome analyses suggest that they mediate their protective effects via different pathways. While pioglitazone downregulates proteins associated with cytoskeleton remodelling, possibly with GSK3β as a regulator of cytoskeleton organization, IL‐4 upregulates proteins involved in protein translation but also calcium homeostasis and reorganization of the actin cytoskeleton (CAMK2D and CAMK2B). Minocycline resulted only in the differential expression of a few proteins. Among the proteins was LAP3, which is involved in glutathione metabolism. This might provide an explanation for the anti‐oxidative function of minocycline in the MitoSox assay (Figure 1N).

### 3.4 Pioglitazone Prevents Glutamate‐Induced Axonal Injury via Inactivation of GSK3β

**FIGURE 3:**
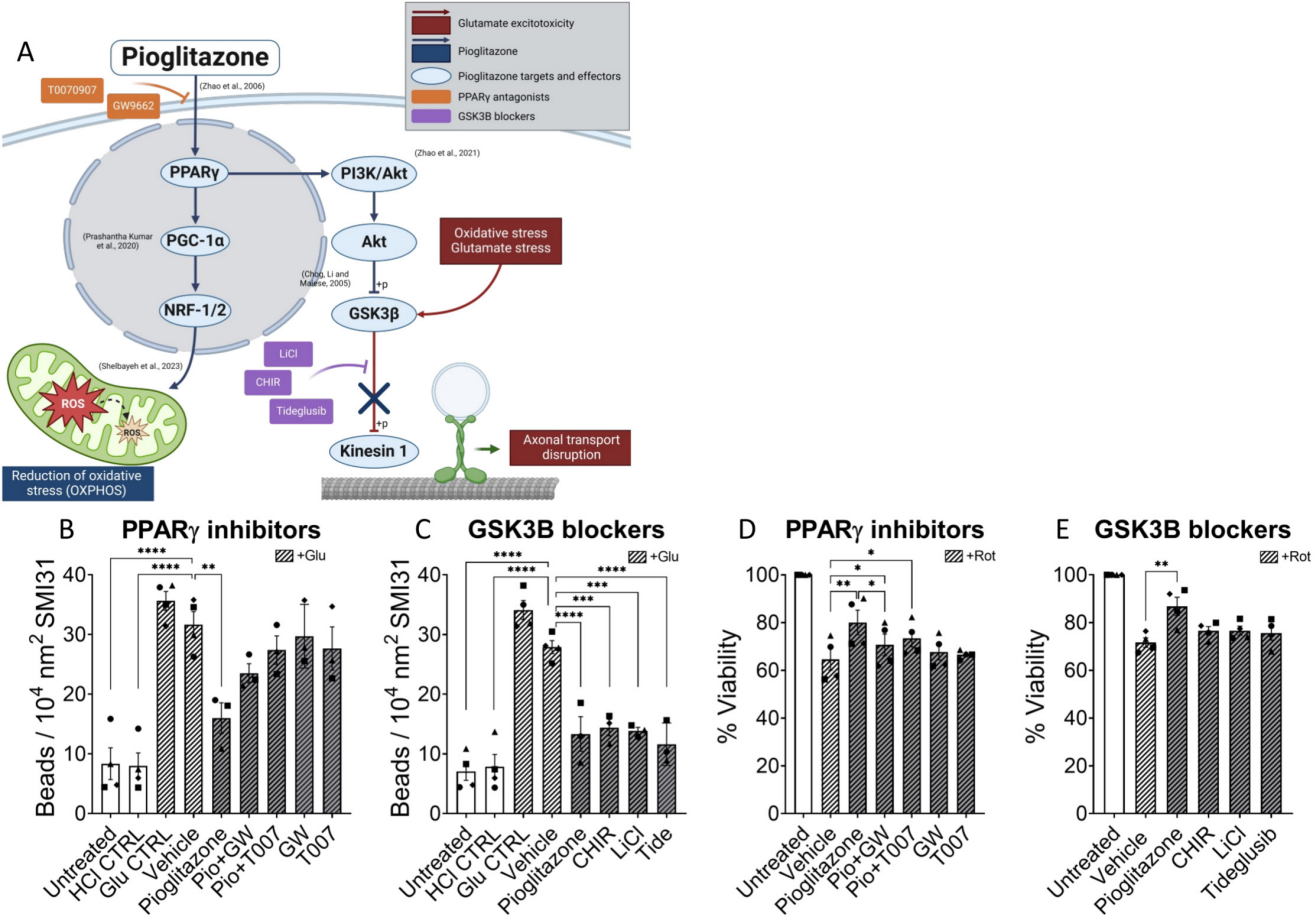
Molecular mechanisms underlying pioglitazone's neuroprotective effects. (A) We propose two distinct pathways by which pioglitazone may inhibit oxidative stress and glutamate‐induced axonal damage. (B) Pioglitazone's reduction of axonal beads upon glutamate input was abrogated when combined with the PPARγ antagonists T007 and GW. (C) In addition, the reduction of axonal beading was recapitulated by the GSK3β blockers CHIR, LiCl and tideglusib. (D) In the context of oxidative stress, PPARγ antagonists T007 and GW partially abrogated pioglitazone's neuroprotective effect. (E) In contrast to the glutamate‐induced axonal damage assay, the GSK3β blockers did not show a beneficial effect on oxidative stress‐induced neuronal loss, as observed in the viability assay. *n* = 3–4 independent experiments *p ≤ 0.05, **p ≤ 0.01, ***p ≤ 0.001 and ****p ≤ 0.0001; one‐way ANOVA followed by Dunnett's multiple comparison test. GW, GW9662; CHIR, CHIR 99021; Pio, pioglitazone; T007, T0070907.

We decided to dissect further, how pioglitazone exerts its neuroprotective effects. Pioglitazone, a PPARγ agonist, has demonstrated neuroprotective properties in models of neuronal injury involving glutamate, NMDA and demyelination (Zhao et al. 2006; Licht‐Mayer et al. 2020). Based on previous work and our own findings, we suggest that this protection is mediated by PI3K and Akt signalling and GSK3β phosphorylation resulting in the prevention of disturbed axonal transport (Chong et al. 2005; Zhao et al. 2021) as well as an upregulation of the PPAR‐γ coactivator‐1α (PGC‐1α) and the nuclear respiratory factors NRF‐1/2, which reduces oxidative stress (Figure 3A) (Licht‐Mayer et al. 2020) (Prashantha Kumar et al. 2020; Abu Shelbayeh et al. 2023).

To test this hypothesis, we conducted axonal damage assays combining pioglitazone with PPARγ antagonists GW9662 and T0070907. The protective effect of pioglitazone was abrogated in the presence of these antagonists, with no additional harm observed from individual inhibitor treatments (Figure 3B). Further experiments involved the testing of three GSK3β inhibitors against glutamate‐induced axonal damage: CHIR 99021, LiCl, and tideglusib. These inhibitors displayed a similar reduction in axonal damage compared to pioglitazone treatment (Figure 3C). In addition, PPARγ antagonists partially abrogated the neuroprotective effect of pioglitazone in rotenone‐induced oxidative stress (Figure 3D). In contrast, GSK3β blockers had no protective effects against neuronal death induced by oxidative stress (Figure 3E). These results underscore the pivotal role of PPARγ activation in pioglitazone's neuroprotection and highlight GSK3β downregulation as a key mechanism against glutamate‐induced axonal damage.

### 3.5 IL‐4, Minocycline and Pioglitazone Promoted the Differentiation of Human iPSC‐Derived Oligodendrocytes

Remyelination represents an endogenous and spontaneous neuroprotective mechanism. A prerequisite for successful remyelination by OPC is the differentiation of these progenitors into mature and myelin sheath‐forming oligodendrocytes. Therefore, we tested the promyelinating properties of the compounds in a differentiation assay using human iPSC‐derived oligodendrocytes (hiOLs). In addition to IL‐4, minocycline and pioglitazone, we also included benztropine and clemastine, which previously have been shown to promote oligodendroglial differentiation in vitro and/or in vivo in rodent animal models (Deshmukh et al. 2013; Mei et al. 2014; Najm et al. 2015).

**FIGURE 4:**
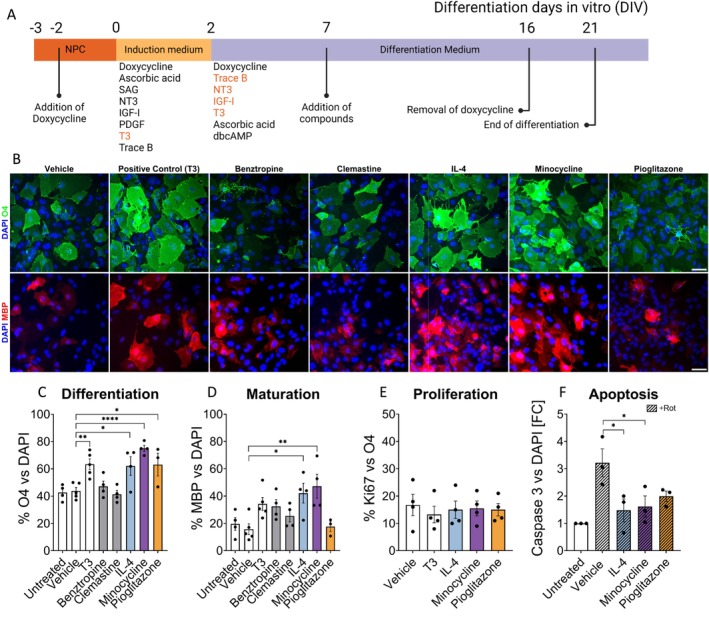
Candidate compound screening with human iPSC‐derived oligodendrocytes. (A) Timeline of the oligodendrocyte differentiation, several small molecules applied with glial induction medium (DIV 0 to DIV 2), and glial differentiation medium (DIV 2 to DIV 21). Minimal medium conditions lack the molecules marked in orange. (B) Representative images of O4+ (upper row) and MBP+ oligodendrocytes (lower row) at the termination of differentiation (DIV 21). (C) Quantifications of O4+ immature oligodendrocytes over DAPI. (D) Quantification of MBP+ mature oligodendrocytes over DAPI. (E) Quantification of Ki67+/O4+ cells, demonstrating the number of proliferating OPCs. (F) Quantification of Caspase‐3+ cells over DAPI, reflecting the anti‐apoptotic feature of the compounds. *n* = 4 independent experiments. Scale bars: 50 um. *p ≤ 0.05, **p ≤ 0.01, ***p ≤ 0.001 and ****p ≤ 0.0001; one‐way ANOVA followed by Dunnett's multiple comparison test.

HiOLs were cultured in minimal medium and treated with either vehicle (0.1% DMSO) as a negative control, triiodothyronine (T3), benztropine and clemastine as positive controls or the drug candidates (Figure 4A). To facilitate the detection of a beneficial effect of the compounds, we cultured the hiOLs in minimal medium as described previously (Ehrlich et al. 2017). As expected, treatment with minimal medium for 21 days decreased the percentage of O4‐positive oligodendrocytes and MBP‐positive mature hiOLs significantly compared to full medium (O4: 42.6% ± 5.7% versus 64.1% ± 11.2%; MBP: 19.5% ± 9.2% versus 41.2% ± 8.9%) (Figure S4B). IL‐4, minocycline and pioglitazone significantly promoted the differentiation of hNPCs into O4+ hiOLs, and IL‐4 and minocycline increased the percentage of MBP+ hiOLs compared to vehicle control (Figure 4B–D). Interestingly, IL‐4 and minocycline both performed better than benztropine or clemastine in this differentiation assay. Furthermore, we tested the effect of IL‐4, minocycline and pioglitazone in a dose‐dependent manner (between 0.01 and 5 μM for minocycline and pioglitazone and between 5 and 100 ng/mL for IL‐4). We observed a bell‐shaped dose–response curve, and maximal effects were achieved at concentrations of 1 μM (minocycline and pioglitazone) or 50 ng/mL (IL‐4), respectively (Figure S4C–E).

To investigate whether the increased numbers of O4+ and MBP+ oligodendrocytes are indeed due to increased differentiation and not mediated by increased proliferation, we performed double ICC for O4 and the proliferation marker Ki‐67 at Day 14 of differentiation. None of the compounds altered the ratio of Ki67+/O4+ cells compared to vehicle control, suggesting that the increased numbers of oligodendrocytes in our cultures were due to the differentiation, not proliferation, of OPCs (Figure 4E). To analyse whether all three compounds protect hiOLs against oxidative stress‐induced cell death as observed for hiNs, hiOLs were treated for 24 h with rotenone starting on Day 18 of differentiation. Cell death was determined by ICC for cleaved Caspase‐3. Rotenone significantly increased the number of cleaved Caspase‐3+ cells. All three compounds reduced the number of cleaved Caspase‐3+ cells; however, only the results for IL‐4 and minocycline were statistically significant (Figure 4F).

In summary, our data demonstrate that all three compounds significantly increased the differentiation of hiOLs suggesting a potentially beneficial effect on remyelination. Furthermore, IL‐4 and minocycline reduced the oxidative stress‐induced cell death of human iPSC‐derived oligodendrocytes.

### 3.6 Pioglitazone Reduces Neuronal Loss and Acute Axonal Damage but Does Not Prevent Oligodendroglial Loss or Demyelination in CPZ‐Induced Demyelination In Vivo

In contrast to IL‐4, pioglitazone and minocycline are FDA‐approved drugs. Thus, we decided to focus on these two compounds in subsequent in vivo experiments. We chose the CPZ model since it results in significant cortical demyelination, neuronal and axonal injury and oligodendroglial loss. To determine the effect of the two compounds on acute axonal damage, we quantified the number of APP+ axonal spheroids; we also counted myeloid and T cells in the corpus callosum. Furthermore, we quantified the numbers of NeuN+ neurons, oligodendrocytes, macrophages/microglia and T cells in the demyelinated cortex.

**FIGURE 5:**
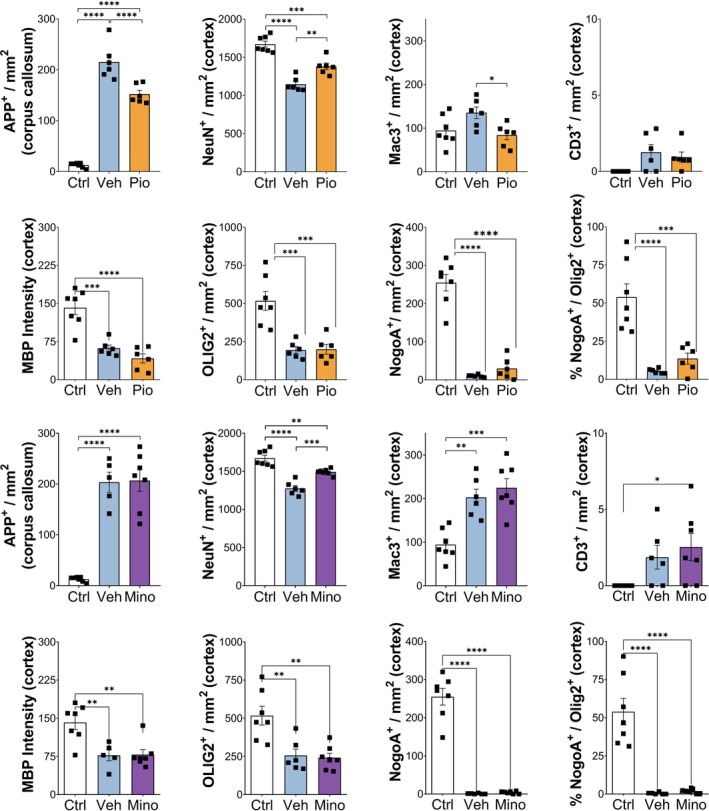
Pioglitazone and minocycline treatment during CPZ‐induced demyelination in mice. (A) Pioglitazone was administered daily (15 mg/kg/day, orally) from Week 4 to Week 6 of CPZ feeding (0.2% in chow). (B) Axonal damage in the corpus callosum was assessed by staining for APP, which accumulates in transected axons. After 6 weeks of CPZ, APP+ spheroids significant increase in number, which was partially reduced in pioglitazone‐treated mice. (C) Similarly, the cortical neuronal loss observed after 6‐week demyelination was reduced in the pioglitazone group. (D) Mac3 staining revealed an increased number of activated macrophages/microglia in the cortex of CPZ‐fed animals, which were reduced upon pioglitazone treatment. The number of (E) T cells, (F) Mbp intensity, (G) Olig2+ oligodendroglial cells, (H) NogoA+ mature oligodendrocytes and (I) the ratio of NogoA+/Olig2+ in the cortex was significantly reduced at 6 weeks of CPZ feeding, but they were not affected by pioglitazone treatment. (I) Minocycline was administered daily (25 mg/kg/day, i.p.) from Week 4 to Week 6 of CPZ feeding (0.2% in chow). (J) Minocycline did not prevent accumulation of APP+ steroids in the corpus callosum. However, (K) it did significantly reduce NeuN+ numbers in the cortex. (L) The number of activated macrophages/microglia remained unchanged upon minocycline treatment, as well as the number of (M) T cells, (N) MBP intensity, (O) Olig2+ oligodendroglial cells, (P) NogoA+ mature oligodendrocytes and (Q) the ratio of NogoA+/Olig2+ in the cortex. *n* = 6–7 animals/group. Data are presented as mean ± SEM statistical significance was determined using one‐way ANOVA with Tukey's multiple comparison test. *p*‐Values < 0.05 were considered significant (*p < 0.05, **p < 0.01, ***p < 0.001 and ****p < 0.0001).

Following 6 weeks of demyelination, we observed a significant increase in the number of APP+ axonal spheroids in the corpus callosum of mice fed with CPZ and treated with vehicle compared to control mice (Figure 5B,J, Figure S5A). Furthermore, in vehicle‐treated CPZ mice compared to control mice, the signal of NeuN+ neurons was reduced (Figure 5C,K, Figure S5B), whereas the number of MAC3+ macrophages/microglia was increased (Figure 5D,L, Figure S5C). Additionally, CPZ treatment resulted in low numbers of CD3+ T cells (Figure 5E,M), loss of MBP+ myelin as well as reduced numbers of OLIG2+ and NOGOA+ oligodendrocytes in the cortex (Figure 5F–I; N–Q, Figure S5D–F).

Treatment with pioglitazone reduced the numbers of APP+ axonal spheroids in the corpus callosum but had no significant effect on T cells or myeloid cells (Figure 5B, Figure S6). In the cortex, pioglitazone treatment was associated with increased numbers of NeuN+ neurons and slightly reduced numbers of MAC3+ myeloid cells compared to vehicle‐treated mice (Figure 5C,D, Figure S5B,C).

Treatment with minocycline resulted in higher numbers of neurons with NeuN immunopositivity but had no effect on the numbers of APP+ axons, MAC3+ myeloid cells and CD3+ T cells, in either the cortex or the corpus callosum (Figure 5J–L, Figures S5–S7). Neither pioglitazone nor minocycline prevented demyelination or the loss of either OLIG2+ or NOGOA+ oligodendrocytes in the cortex (Figure 5 F–I; N–Q, Figure S5D–F).

In summary, our data demonstrate that pioglitazone and minocycline both have neuroprotective effects in a demyelinating animal model. Both compounds reduce the loss of NeuN signal in CPZ‐induced cortical demyelination. Furthermore, pioglitazone, but not minocycline, reduced the number of APP+ axonal spheroids in the corpus callosum. However, both compounds had no effect on oligodendroglial loss or demyelination.

## 4 Discussion

Here, we present an in vitro platform to test the direct neuroprotective and potential promyelinating effects of selected candidate compounds in human iPSC‐derived neurons and oligodendrocytes in a proof of concept study. Using various in vitro assays, we identified several compounds with direct neuroprotective properties without negatively affecting oligodendrocytes. Proteomic analyses revealed unique pathways activated by these compounds, suggesting new modes of action and potential pharmacological targets. Additionally, we tested the neuroprotective properties of pioglitazone and minocycline in vivo.

We generated human iPSC‐derived neurons, which showed a ganglia‐like structure of the neuronal cell bodies and a dense, homogenously distributed neurofilament‐H network. Although we used a protocol that in the past resulted in the generation of midbrain neurons, our neuronal population consisted mostly of glutamatergic excitatory neurons, and to a lesser extent of GABAergic inhibitory and dopaminergic neurons resembling the composition of the human brain cortex (Reinhardt et al. 2013; Lodato and Arlotta 2015).

The initial step in developing this platform involved selecting pathophysiologies relevant to neurodegeneration in MS and establishing cell culture assays mimicking these conditions. We focused on neuronal loss and axonal injury. Neuronal loss in the cortex and basal ganglia is prominent in MS and extensive cortical neuronal loss correlates with cortical volume and a more severe disease course (Magliozzi et al. 2010; Carassiti et al. 2018). Acute axonal injury is a typical feature of early MS lesion stages and is associated with inflammation (Trapp et al. 1998; Kuhlmann et al. 2002). Acute axonal injury is characterized by the formation of so‐called axonal spheroids and although some of them might be reversible at least in animal models of MS, frequently they are associated with transected axons (Nikic et al. 2011). Acute axonal injury and subsequent Wallerian degeneration contribute significantly to axonal loss, which correlates with clinical disability in MS (Tallantyre et al. 2010). Axonal density is reduced by 60% throughout the spinal cord over a time period of 30 years (Tallantyre et al. 2010; Petrova et al. 2018). However, the exact molecular mechanisms resulting in neuronal loss and acute axonal injury in MS are only partially understood. Multiple studies suggest that mitochondrial injury as well as activation of glutamate receptors plays a key role in axonal and neuronal injury and loss in MS. Mitochondrial dysfunction results in an energy deficit, impaired electron transport chain function and increased ROS production, whereas activation of glutamate receptors is associated with calcium influx, which in turn activates a number of Ca^2+^‐dependant enzymes, such as proteases resulting in axonal degeneration (Lau and Tymianski 2010; Calabrese et al. 2015; Woo et al. 2024). In rodent cell cultures, the addition of glutamate triggers cytoskeletal degeneration and formation of axonal beads similar to the changes observed in our human neuronal cultures after exposure to glutamate (Reynolds et al. 2011).

To mimic these aspects of the disease we used rotenone, a complex I inhibitor of the mitochondrial respiratory chain resulting in reduced ATP generation and increased ROS production, and glutamate, to induce acute axonal injury. We then carried out further analyses of compounds that had shown direct neuroprotective effects in both assays. Subsequent assays were performed to validate the neuroprotective effects of candidate compounds and to exclude any that might have detrimental effects on oligodendrocytes, the other major target population in MS.

Amiloride and Edaravone both had no effect on axonal damage or on rotenone‐induced neuronal loss in our in vitro studies and were therefore excluded from further analyses. All three remaining compounds, IL‐4, minocycline and pioglitazone protected against glutamate‐induced axonal injury and rotenone‐induced cell death and the protective effect against cell death was supported by additional in vitro assays. IL4 binds to IL4Ra‐containing receptors and activates the JAK‐STAT6 pathway in immune cells (Kaplan et al. 1996). Besides its well‐known immunomodulatory functions, IL‐4 reversed disease progression in chronic EAE without affecting inflammation and reduced excitotoxic cell death as well as neurite outgrowth in primary rodent neurons, which is in line with our observations (Payne et al. 2012; Walsh et al. 2015; Vogelaar et al. 2018). Furthermore, in our study, IL‐4 also reduced glutamate‐induced axonal injury and oligodendroglial cell death and it increased oligodendroglial differentiation. These combined immunomodulatory as well as neuroprotective and potentially promyelinating effects make IL‐4 an interesting candidate for future studies. Previous studies suggest that IL‐4 exerts its neuroprotective effects via phosphorylation of PI3K and GAP‐43 and reduced calmodulin binding, which are involved in the modification of the actin cytoskeleton. Our proteomic analyses support this notion, since we observed the upregulation of proteins belonging to the GO terms ‘calcium‐ and calmodulin‐dependent protein kinase complex’. However, we also observed the upregulation of proteins belonging to the GO terms ‘translational initiation’, ‘spliceosomal complex’ and ‘chromatin silencing’, suggesting that additional molecular mechanisms may contribute to the neuroprotective effects mediated by IL‐4.

For the anti‐biotic minocycline, which binds to the 30s ribosomal subunit of bacteria and thus inhibits protein synthesis (Asadi et al. 2020), additional anti‐inflammatory, anti‐oxidant and anti‐apoptotic effects have been described (Yong et al. 2004). In clinical MS trials, minocycline reduced the number of enhancing lesions and delayed the conversion from clinically isolated syndrome to definitive MS, at least temporarily, pointing to an anti‐inflammatory effect of minocycline. This assessment is further supported by in vivo animal studies (Metz et al. 2004; Cunha 2006; Metz et al. 2017; Faissner et al. 2018). However, anti‐oxidant and anti‐apoptotic effects have also been described (Wilkins et al. 2004; Kraus et al. 2005). In line with that, minocycline also reduced the loss of NeuN+ signal in vivo without affecting inflammation, strengthening the possibility that minocycline also exerts in vivo a direct neuroprotective effect. However, despite encouraging effects in vitro, minocycline did not reduce oligodendroglial loss or demyelination. Since the focus of our study was on the neuroprotective effects of the investigated compounds, we did not further investigate potential consequences for remyelination in our in vivo model, which is a limitation of our study. A previous study reported negative consequences of minocycline on remyelination in ethidium bromide‐induced demyelination and suggested that minocycline impaired microglial activation, thereby suppressing remyelination (Li et al. 2005). The molecular mechanisms by which minocycline exerts its neuroprotective effects are unknown. In the proteome analyses, only a few differentially regulated proteins were detected, among them LAP3. LAP3 is suggested to be involved in glutathione metabolism and therefore may play a role in the cellular redox state (Cappiello et al. 2004). This could explain why minocycline treatment led to a significant decrease in ROS production, in contrast to all other compounds. We also identified a possible role for CDKN1B, a cyclin‐dependent kinase inhibitor that, in the cytoplasm, associates with microtubules and is required for proper axonal transport, neuronal migration and dendritic spine formation (Garcia‐Osta et al. 2022). Two other differentially regulated proteins were PKL and MTTP, both of which are involved in metabolic pathways, which might suggest an attempt to generate ATP by alternative sources, to counteract the energy deficit induced by glutamate‐induced excitotoxicity.

Pioglitazone binds to the nuclear receptor PPARγ, thereby altering the transcription of metabolic and inflammatory genes. PPARγ forms heterodimers with retinoid X receptor (RXR) and bexarotene, an RXR agonist, promotes oligodendroglial differentiation and remyelination in rodent animal models (Huang et al. 2011). Pioglitazone is an anti‐diabetic medication that reduces the incidence of EAE and ameliorates its symptoms by suppressing T‐cell activation and decreasing expression of pro‐inflammatory genes (Feinstein et al. 2002). Furthermore, pioglitazone reduced lesion burden and the number of newly developed lesions in MS patients (Kaiser et al. 2009; Shukla et al. 2010; Negrotto et al. 2016). However, whether this was due to the direct anti‐inflammatory effects of pioglitazone or mediated via changes in metabolism remains open for discussion. In vitro, pioglitazone promotes mitochondrial functions and protects acutely demyelinated murine axons from degeneration (Gray et al. 2012; Licht‐Mayer et al. 2020). In our in vitro experiments, pioglitazone reduced glutamate‐induced axonal injury as well as oxidative stress‐induced cell death, thus confirming the direct neuroprotective characteristics of pioglitazone also in human iPSC‐derived neurons. Our proteome studies indicated, in line with previous studies, an involvement of mitochondrial transport as well as cytoskeleton reorganization in the neuroprotective effects of pioglitazone. They also suggested an involvement of GSK3β, and, based on our in vitro studies, we propose two independent modes of action underlying pioglitazone's neuroprotective effects: (1) reduction of mitochondrial ROS via activation of PPARγ and increased expression of PGC‐1α and coactivation of transcription factors (NRF‐1/2); and (2) prevention of axonal transport disruption via phosphorylation of GSK3β by Akt, preventing the former from phosphorylating microtubule‐associated proteins, such as kinesin I (Chong et al. 2005; Rippin and Eldar‐Finkelman 2021; Zhao et al. 2021). In the CPZ model, pioglitazone treatment correlated with slightly increased NeuN+ neuronal densities in the cortex and decreased numbers of APP+ axonal spheroids in the corpus callosum compared to vehicle‐treated mice. However, this was also associated with slightly reduced numbers of T cells and myeloid cells in the corpus callosum or cortex; therefore, we cannot exclude that these neuroprotective effects observed in vivo were mediated indirectly via anti‐inflammatory mechanisms (Yilmaz et al. 2023). Interestingly, pioglitazone neither increased the differentiation of hiOL into mature MBP+ oligodendrocytes, nor did it protect significantly against rotenone‐induced cell death, suggesting that pioglitazone has no strong effect on human oligodendrocytes. This is in contrast to impacts seen on primary rat oligodendroglial cultures and may indicate species differences (Cai et al. 2018; Zhang et al. 2019; De Nuccio et al. 2020).

Our proof of concept study demonstrates the feasibility of our human preclinical platform for testing neuroprotective properties and assessing effects on oligodendroglial differentiation and cell death. Several compounds exhibited beneficial effects in vitro, not only on neurons but also on oligodendrocytes; however, these beneficial effects in vitro did not necessarily translate into protective effects in vivo, suggesting that further optimization and more rigorous in vitro testing are desirable. As previously mentioned, multiple neurodegenerative pathophysiologies contribute to disease progression in MS. In our platform, we focused on two key mechanisms: axonal injury and neuronal loss. Future studies should include assays examining synaptic function and loss, as these may significantly contribute to disease progression (Jurgens et al. 2016; Mock et al. 2021; Huiskamp et al. 2022). The incorporation of further functional readouts, such as electrophysiological analyses, would likewise be advantageous. However, complex phenotypic assays limit the feasibility of higher throughput screens, and the known variability associated with the iPSC lines poses additional challenges for large‐scale drug screenings. Another limitation is the absence of an inflammatory milieu akin to that in MS. This could be addressed by incorporating CSF from MS patients or supernatants from inflammatory cells, such as PBMCs or myeloid cells (Fagiani et al. 2024). However, extensive studies will be required to verify whether this approach results in cellular and molecular changes comparable to those in MS lesions and is therefore out of the focus of this study. An additional challenge is the in vivo validation of compounds successfully tested in vitro. For in vivo validation, the cuprizone model, though widely used for studying MS‐related pathologies, differs from human disease, as demyelination is toxin‐induced and monophasic rather than autoimmune‐induced and chronic (Klotz et al. 2023). Furthermore, distinguishing direct neuroprotective effects from those secondary to reduced inflammation remains challenging, since inflammation and neurodegeneration are closely interlinked also in this model.

## 5 Conclusions

Our study demonstrates that our human preclinical platform can be used to test neuroprotective and promyelinating properties of compounds. Both, minocycline and pioglitazone, showed neuroprotective effects in vitro and in vivo. The use of such human preclinical platforms may facilitate the identification of compounds for future clinical trials aimed at the prevention of disease progression in MS.

## Author Contributions

**Raquel Guerrero González:** formal analysis, investigation, methodology, writing – original draft. **Elif Nur Yilmaz:** investigation, methodology. **Stefanie Albrecht:** formal analysis, investigation, methodology, project administration, writing – original draft, writing – review and editing. **Maurine Fucito:** methodology, validation. **Damiana Pieragostino:** resources, supervision. **Alina Schmidt:** investigation. **Simone König:** data curation, formal analysis. **Una FitzGerald:** funding acquisition, supervision, writing – review and editing. **Tanja Kuhlmann:** conceptualization, funding acquisition, project administration, resources, supervision, writing – original draft, writing – review and editing.

## Ethics Statement

Animal handling and experiments were performed according to the German Animal Welfare Act and approved by the responsible governmental authorities (LANUV Nordrhein‐Westfalen; 81‐02.04.2020.A365). The study was approved by the Ethics Committee of the University of Münster (AZ 2016‐026‐f‐S, AZ 2017‐230‐f‐S).

## Conflicts of Interest

T.K. receives research funding from the German Research Foundation, Interdisciplinary Center for Clinical Research (IZKF) Münster, National MS Society, German MS Society and Novartis. She received compensation for serving on scientific advisory boards from Novartis, Sanofi and Merck and speaker honoraria from Novartis, Biogen, Sanofi and Roche.

## Supporting information


**Figure S1:** Immunocytochemistry of iPSC‐derived NPCs for the neuronal progenitor markers NESTIN and SOX1. Merge picture: NESTIN is depicted in green, SOX1 in red and nuclei are depicted in blue (DAPI). Scale bar equal 100 μm.
**Figure S2:** In vitro assays to assess neuroprotection.
**Figure S3:** Proteomic analysis volcano plots.
**Figure S4:** (A) Immunocytochemistry of iPSC‐derived oligodendrocytes of full and minimal conditions at 21 DIV for immature (O4) and mature (MBP) oligodendrocytes. Nuclei are depicted in blue (DAPI). (B) Comparison of full and minimal (MM) conditions for O4+, MBP+ cells and MBP/O4 ratio. Dose‐dependent effect of (C) IL‐4, (D) minocycline, and (E) pioglitazone on the maturation of oligodendrocytes. Scale bar equals 50 μm. Scale bar equal 50 μm. The level of significance is indicated as: *p ≤ 0.05, **p ≤ 0.01, ***p ≤ 0.001, ****p ≤ 0.0001. Differences were determined using unpaired t‐test.
**Figure S5:** Representative pictures of macrophages/microglia and oligodendrocyte lineage cells in the of CPZ mice treated with pioglitazone or minocycline (A) After demyelination, axonal damage in the corpus callosum was assessed by significant increase of APP+ spheroids, which was partially reduced in pioglitazone‐treated mice but minocycline did not prevent accumulation of APP+ sheroids. (B) The cortical neuronal loss observed after 6 weeks demyelination was reduced in the pioglitazone and minocycline group. (C) Mac3 staining revealed an increased number of activated macrophages/microglia in the cortex of CPZ‐fed animals which were reduced upon pioglitazone treatment but not by minocycline. The (D) Mbp intensity, (E) number of Olig2+ oligodendroglial cells, and (F) NogoA+ mature oligodendrocytes, in the cortex was significantly reduced at 6 weeks of CPZ feeding, but they were not affected by either pioglitazone or minocycline treatment. Scale bars: panels A:50 μm B‐F: 20 μm.
**Figure S6:** Quantification of macrophages/microglia and oligodendrocyte lineage cells in the corpus collosum of CPZ mice treated with pioglitazone or minocycline.
**Figure S7:** Representative pictures of macrophages/microglia and oligodendrocyte lineage cells in the corpus callosum of CPZ mice treated with pioglitazone or minocycline.

**Data S1:** Supporting information.

## Data Availability

The datasets used and analysed during the current study are available from the corresponding author on reasonable request.
