# Supplementary material for: Development of a Human Preclinical Platform for the Identification of Neuroprotective Compounds
Source: Eur J Neurosci. 2025 Nov 23;62(10):e70328. doi: 10.1111/ejn.70328 (PMC12641211; doi:10.1111/ejn.70328)
Supplement: Supplementary file 1 — Figure S1: Immunocytochemistry of iPSC‐derived NPCs for the neuronal progenitor markers NESTIN and SOX1. Merge picture: NESTIN is depicted in green, SOX1 in red and nuclei are depicted in blue (DAPI). Scale bar equal 100 μm. Figure S2: In vitro assays to assess neuroprotection. Figure S3: Proteomic analysis volcano plots. Figure S4: (A) Immunocytochemistry of iPSC‐derived oligodendrocytes of full and minimal conditions at 21 DIV for immature (O4) and mature (MBP) oligodendrocytes. Nuclei are depicted in blue (DAPI). (B) Comparison of full and minimal (MM) conditions for O4+, MBP+ cells and MBP/O4 ratio. Dose‐dependent effect of (C) IL‐4, (D) minocycline, and (E) pioglitazone on the maturation of oligodendrocytes. Scale bar equals 50 μm. Scale bar equal 50 μm. The level of significance is indicated as: *p ≤ 0.05, **p ≤ 0.01, ***p ≤ 0.001, ****p ≤ 0.0001. Differences were determined using unpaired t‐test. Figure S5: Representative pictures of macrophages/microglia and oligodendrocyte lineage cells in the of CPZ mice treated with pioglitazone or minocycline (A) After demyelination, axonal damage in the corpus callosum was assessed by significant increase of APP+ spheroids, which was partially reduced in pioglitazone‐treated mice but minocycline did not prevent accumulation of APP+ sheroids. (B) The cortical neuronal loss observed after 6 weeks demyelination was reduced in the pioglitazone and minocycline group. (C) Mac3 staining revealed an increased number of activated macrophages/microglia in the cortex of CPZ‐fed animals which were reduced upon pioglitazone treatment but not by minocycline. The (D) Mbp intensity, (E) number of Olig2+ oligodendroglial cells, and (F) NogoA+ mature oligodendrocytes, in the cortex was significantly reduced at 6 weeks of CPZ feeding, but they were not affected by either pioglitazone or minocycline treatment. Scale bars: panels A:50 μm B‐F: 20 μm. Figure S6: Quantification of macrophages/microglia and oligodendrocyte lineage cells in the [file EJN-62-0-s001.pdf]

SUPPLEMENTARY FIGURES

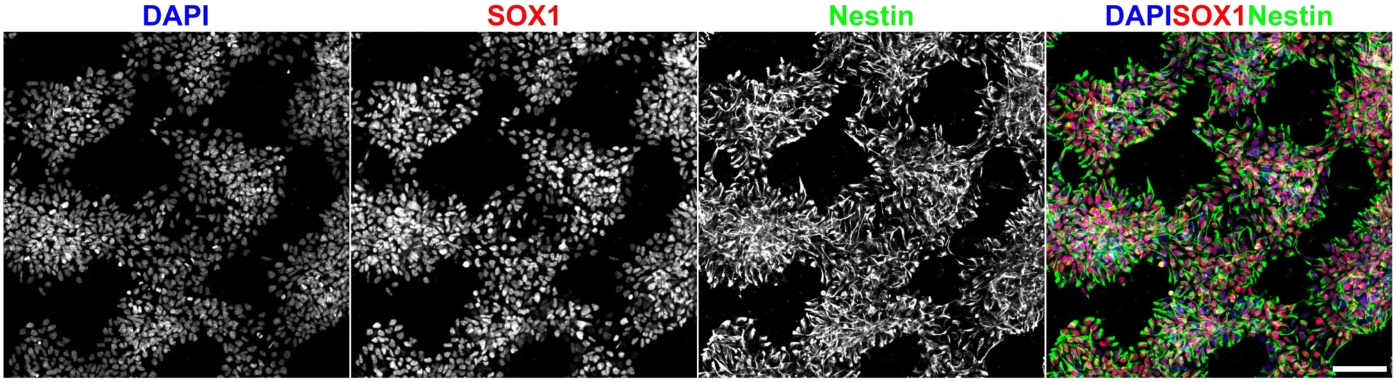

Supplementary Figure 1. Immunocytochemistry of iPSC-derived NPCs for the neuronal progenitor markers NESTIN and SOX1. Merge picture: NESTIN is depicted in green, SOX1 in red and nuclei are depicted in blue (DAPI). Scale bar equal 100 µm.

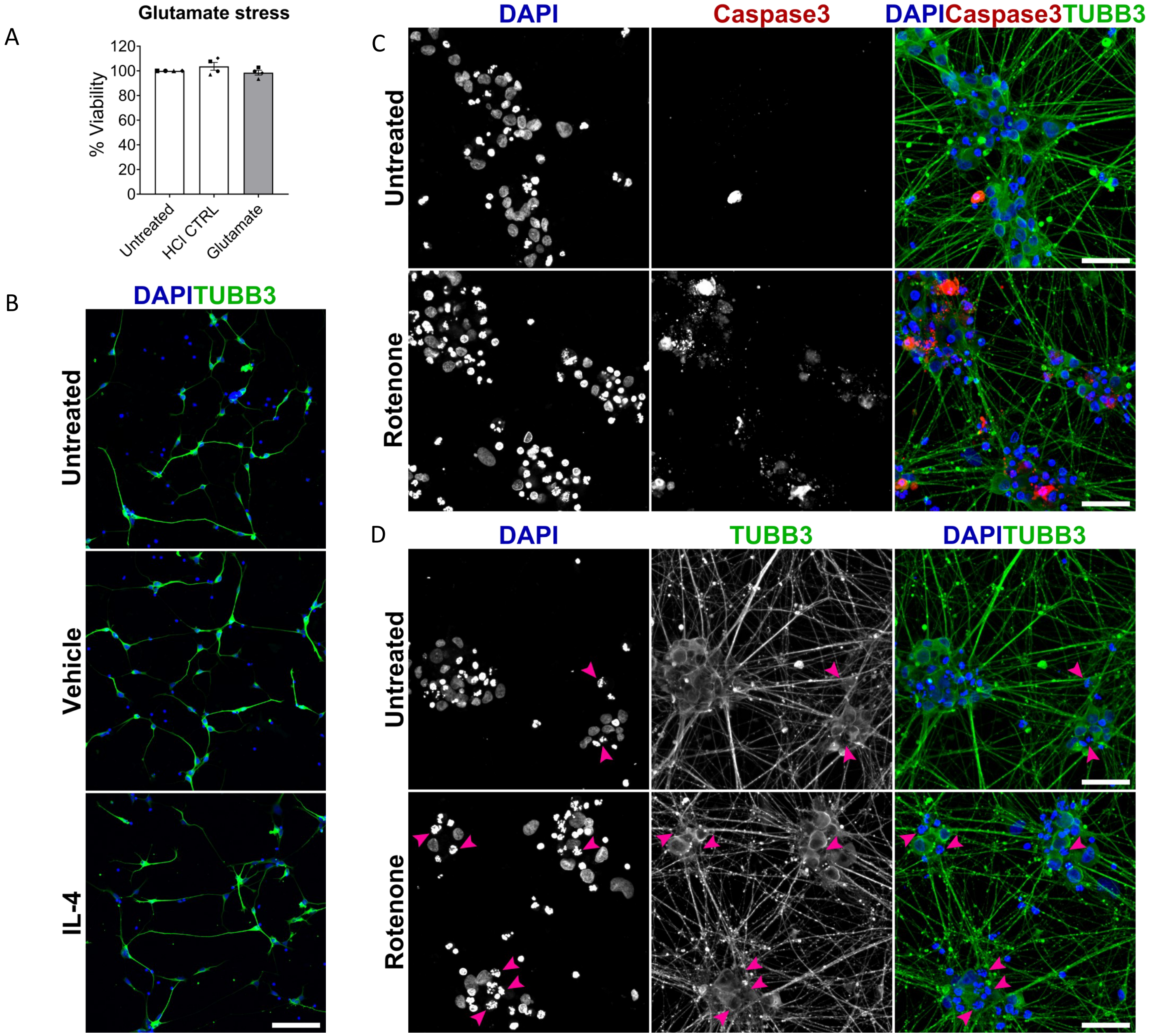

Supplementary Figure 2. ***In vitro*** **assays to assess neuroprotection.**

(A) Glutamate stress did not induce cell death. CellTiterGlo Assay showed no impaired cell viability due to glutamate treatment used for axonal damage induction (1 mM of glutamate for 24 h).

(B) For assessing neurite outgrowth, cells at 10 days *in vitro* (DIV) were replated and treated with Minocycline, Pioglitazone or IL-4 for 24 hours. At 11 DIV, the cells were fixed and stained for TUBB3. Neurite length was determined by measuring the longest neurite of each TUBB3-positive neuron using the NeuronJ plugin in ImageJ. Only IL-4 significantly increased neurite length. Scale bar equal 100 µm.

(C) Treatment with rotenone (1 µM for 6 hours at 22 DIV) resulted in an elevation of Caspase3-positive neurons (TUBB3-positive).

(D) Additionally, as an indicator of apoptotic cells, neurons with fragmented nuclei (indicated by arrowheads) were quantified following rotenone treatment (1 µM for 6 hours at 22 DIV). Scale bar equal 50 µm.

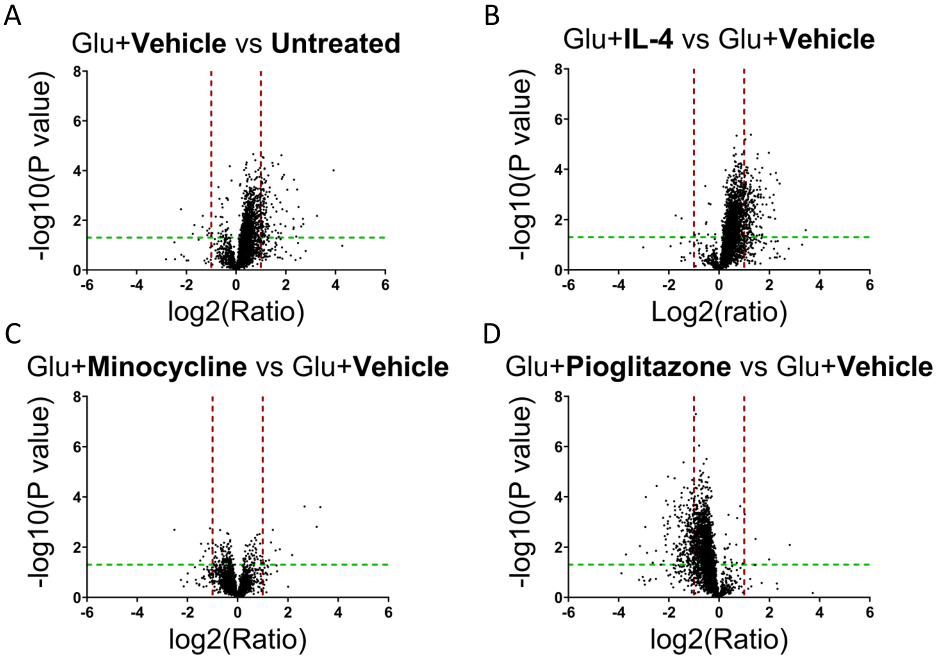

Supplementary Figure 3. Proteomic analysis volcano plots

Volcano plot of differentially expressed proteins in **(A)** vehicle-treated neurons exposed to glutamate versus untreated neurons, and **(B)** IL-4-treated neurons, **(C)** minocycline-treated neurons or **(D)** pioglitazone-treated neurons compared to vehicle-treated neurons exposed to glutamate.

Values above the green dotted line are considered statistically significant (p value < 0.05; indicated by -log10(P value) = 1.3 in the graph), while the red dotted lines mark the chosen fold change threshold for down (left, log2(Ratio) = -1) and upregulated (right, log2(Ratio) = 1) proteins.

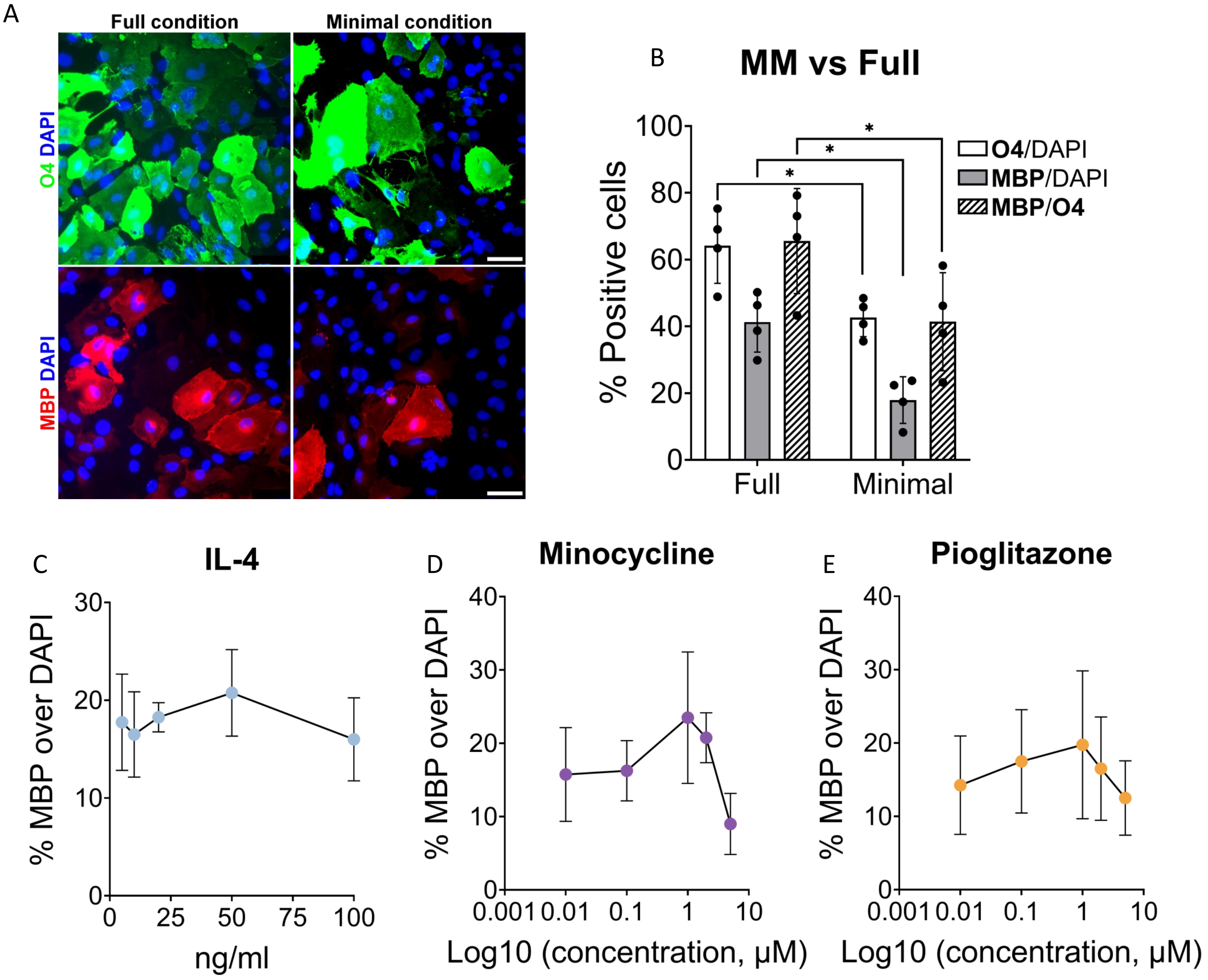

Supplementary Figure 4.

(A) Immunocytochemistry of iPSC-derived oligodendrocytes of full and minimal conditions at 21 DIV for immature (O4) and mature (MBP) oligodendrocytes. Nuclei are depicted in blue (DAPI). **(B)** Comparison of full and minimal (MM) conditions for O4+, MBP+ cells and MBP/O4 ratio. Dose-dependent effect of **(C)** IL-4, **(D)** minocycline, and **(E)** pioglitazone on the maturation of oligodendrocytes. Scale bar equals 50 µm. Scale bar equal 50 µm. The level of significance is indicated as: *p ≤ .05, **p ≤ .01, ***p ≤ .001, ****p ≤ .0001. Differences were determined using unpaired t-test.

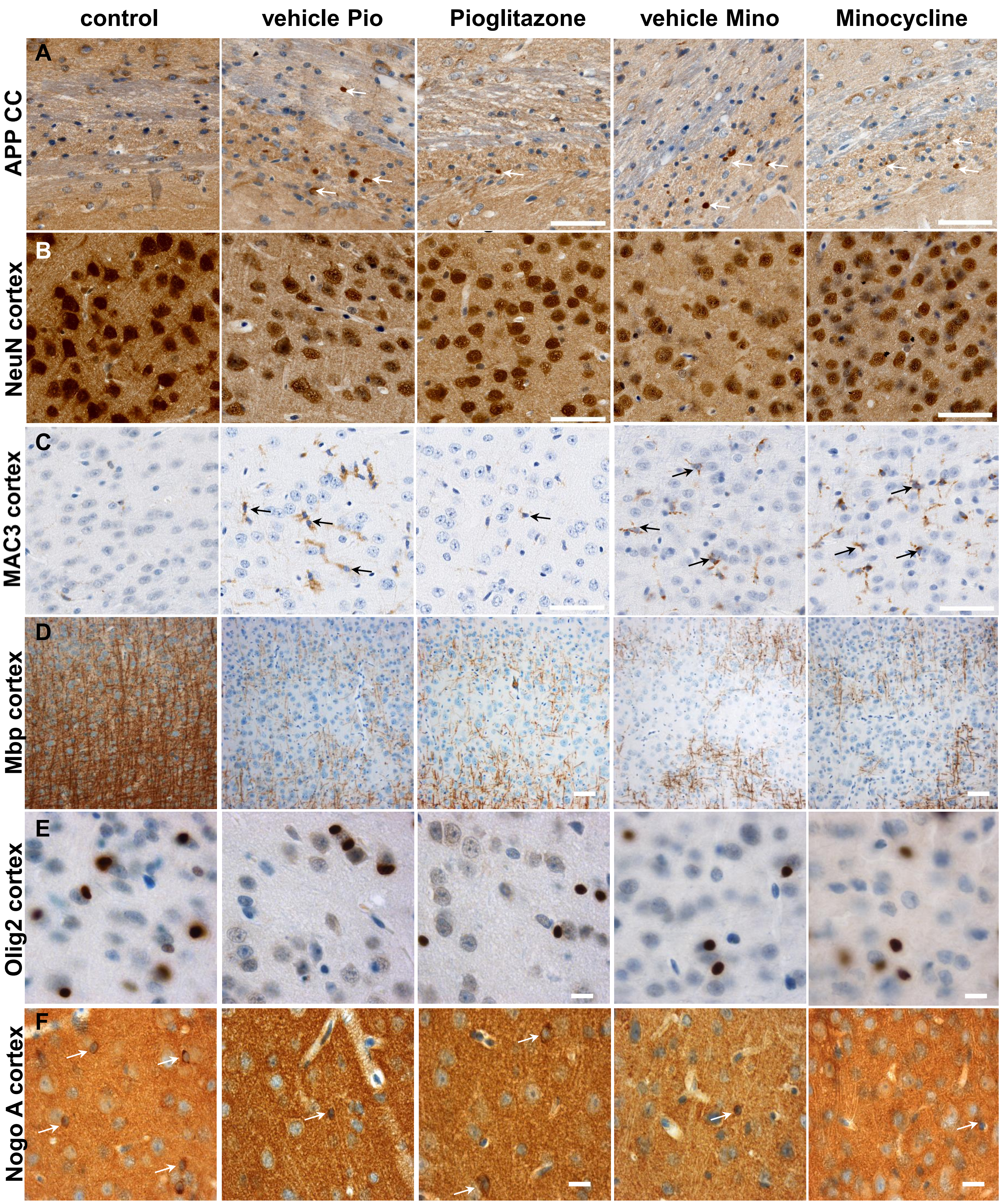

Supplementary Figure 5. Representative pictures of macrophages/microglia and oligodendrocyte lineage cells in the of CPZ mice treated with pioglitazone or minocycline

(A) After demyelination, axonal damage in the corpus callosum was assessed by significant increase of APP+ spheroids, which was partially reduced in pioglitazone-treated mice but minocycline did not prevent accumulation of APP+ sheroids. **(B)** The cortical neuronal loss observed after 6 weeks demyelination was reduced in the pioglitazone and minocycline group. **(C)** Mac3 staining revealed an increased number of activated macrophages/microglia in the cortex of CPZ-fed animals which were reduced upon pioglitazone treatment but not by minocycline. The **(D)** Mbp intensity, **(E)** number of Olig2+ oligodendroglial cells, and **(F)** NogoA+ mature oligodendrocytes**,** in the cortex was significantly reduced at 6 weeks of CPZ feeding, but they were not affected by either pioglitazone or minocycline treatment. Scale bars: panels A:50 µm B-F: 20µm.

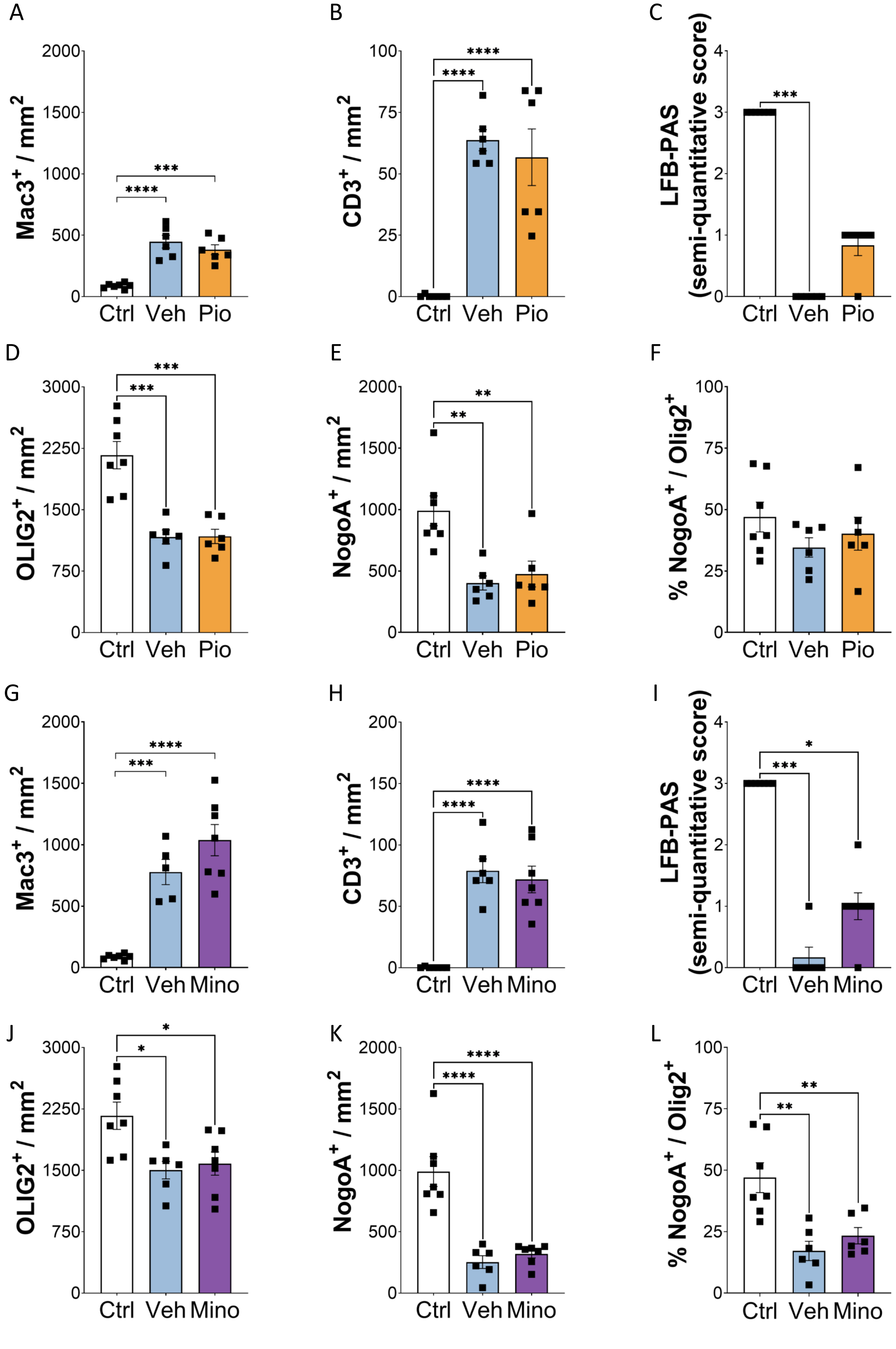
A
B
C
D
E
F
G
H
I
J
K
L
Mac3+ / mm2
CD3+ / mm2
LFB-PAS (semi-quantitative score)
OLIG2+ / mm2
NogoA+ / mm2
% NogoA+ / Olig2+
Ctrl
Veh
Pio
Mino

Supplementary Figure 6. Quantification of macrophages/microglia and oligodendrocyte lineage cells in the corpus collosum of CPZ mice treated with pioglitazone or minocycline

(A) There was a significant increase of infiltrated macrophages/microglia at 6 weeks of demyelination compared to the healthy control in the corpus callosum. However, pioglitazone treatment had no significant effect in the number of Mac3+ cells. **(B)** the same was observed for the number of infiltrated T cells. **(C)** Pioglitazone had no effect on the extent of myelination, as measured by LFB-PAS semi-quantification. Pioglitazone did not alter the number of oligodendroglial cell numbers, as seen by **(D)** OLIG2+ cells, **(E)** NogoA+ mature oligodendrocytes, and **(F)** the NogoA+/OLIG2+ ratio.

Similarly, minocycline did not affect the number of **(G)** Mac3+ cells nor **(H)** infiltrated T cells in the corpus callosum at the end of 6 weeks demyelination. (I) LFB-PAS semi-quantification remained unchanged. Oligodendrocyte numbers were also not affected, as quantified by **(J)** OLIG2+ cells, **(K)** NogoA+ cells and **(L)** OLIG2+/NogoA+ ratio.

Data is presented as mean ± SEM. Statistical significance was determined using one-way ANOVA with Tukey’s multiple comparison test, except for the LFB-PAS semi-quantification, where a Kruskal-Wallis test with Dunn`s multiple comparison was performed. P-values<0.05 were considered significant (*p <0.05, **p <0.01, ***p <0.001, ****p ≤ .0001).

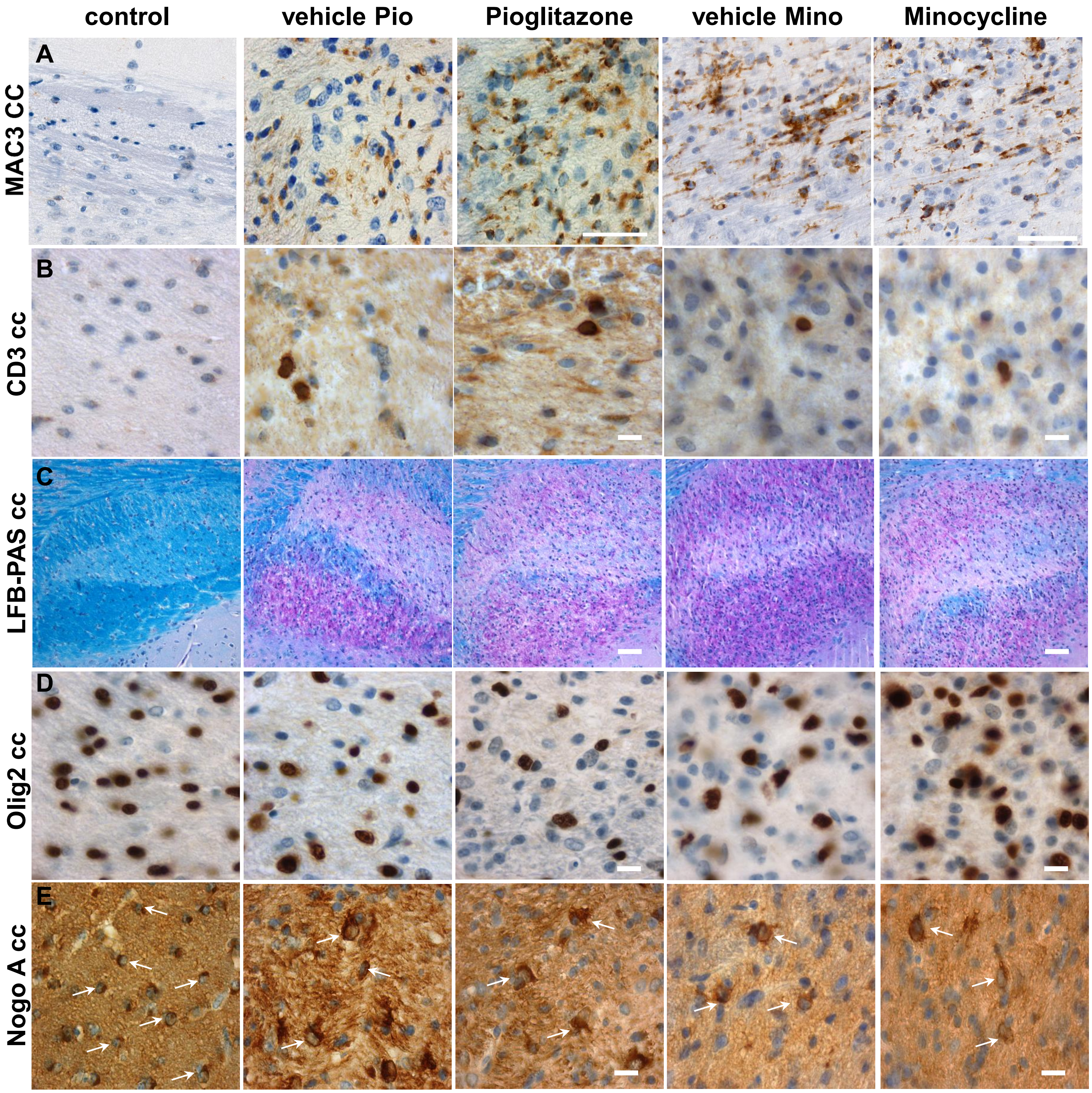

Supplementary Figure 7. Representative pictures of macrophages/microglia and oligodendrocyte lineage cells in the corpus collosum of CPZ mice treated with pioglitazone or minocycline

(A) Infiltrated Mac3+ macrophages/microglia at 6 weeks of demyelination compared to the healthy control in the corpus callosum. Pioglitazone or minocycline treatment had no significant effect in the number of Mac3+ cells. **(B)** the same was observed for the number of infiltrated CD3+ T cells. **(C)** Pioglitazone and minocycline had no effect on the extent of myelination, as measured by LFB-PAS semi-quantification. Pioglitazone did not alter the number of oligodendroglial cell numbers, as seen by **(D)** OLIG2+ cells, **(E)** NogoA+ mature oligodendrocytes. Scale bars: panel A: 50 µm, B-E: 20µm
