# Supplementary material for: Development of a Human Preclinical Platform for the Identification of Neuroprotective Compounds
Source: Eur J Neurosci. 2025 Nov 23;62(10):e70328. doi: 10.1111/ejn.70328 (PMC12641211; doi:10.1111/ejn.70328)
Supplement: Supplementary file 2 — Data S1: Supporting information. [file EJN-62-0-s002.pdf]

Supplementary Table: Primary and secondary antibodies used for immunocytochemistry

**Name**	**Company, Cat. No.; Dilution**
**Primary Antibodies**	
Mouse anti-Tubulin β3	Biolegend, 801201; 1:1000
Mouse anti-APP	Millipore, MAB348; 1:2000
Rabbit anti-cleaved Caspase-3 (Asp175)	Cell Signaling, 9661; 1:1000
Rat anti-CD3	Serotec, MCA1477; 1:100
Rabbit anti-GABA	Sigma, A2052; 1:100
Rabbit anti-vGlutT1	Abcam, ab227805; 1:150, 1:2000 IHC
Rabbit anti-Ki67	Abcam, AB16667, 1:100
rat anti-Mac3	BD, 553322; 1:200
Rat anti-MBP	Abcam, AB7349, 1:4000
Mouse anti-NESTIN	R&D systems, MAB1259, 1:300
mouse anti-NeuN	Chemicon, MAB377; 1:100
Mouse anti-Neurofilament H (NF-H), Phosphorylated (SMI-31)	Biolegend, 801601; 1:1000
mouse anti-NOGOA (Clone 11C7)	Generous gift from M.E. Schwab, Brain Research Institute, University of Zürich and Department of Biology, Swiss Federal Institute of Technology Zürich, Switzerland 1:20,000
Mouse anti-O4	R&D systems, AB1326, 1:100
Rabbit anti-OLIG2	IBL, 18953; 1:150
Goat anti-Sox1	R&D systems, AF3369, 1:150
Rabbit anti-TH	Santa Cruz, sc-14007; 1:500
**Secondary Antibodies**	
Alexa Fluor 488 anti-mouse (Goat)	Jackson/Dianova, 115-545-166; 1:250
Alexa Fluor Cy3 anti-rabbit (Goat)	Jackson/Dianova, 111-165-144; 1:250
Cyc3 anti-goat (Donkey)	Jackson/Dianova, 705-165-147; 1:250

Supplementary Table 2 (Excel File). Down and upregulated proteins obtained from proteomic analysis of iPSC-derived neurons upon glutamate treatment and treatment with the compounds. Proteins were considered downregulated when t ratio ≤ 0.5; and upregulated when t ratio ≥ 2.

Supplementary Table 3 Down and upregulated proteins found in the pathway enrichment of the proteomic analysis of iPSC-derived neurons exposed to glutamate and treated with the compounds. Pathways were considered significant when p-value < 0.05. Pathways from KEGG Human 2021 database.

**Term**	**Overlap**	**P-value**	**Proteins**
**Glu+Vehicle vs Untreated: Upregulated**			
Axon guidance	11/182	2,33E-08	ENAH CDC42 EPHA4 DPYSL5 CFL1 PLXNA1 FYN KRAS EPHB2 CAMK2G GNAI1
Cholinergic synapse	8/113	6,20E-07	GNAO1 GNG2 GNB1 GNB4 FYN KRAS CAMK2G GNAI1
Endocytosis	10/252	4,85E-06	CDC42 RAB10 CYTH2 RAB5B IST1 IQSEC1 RAB35 WASHC2C RAB8A ARF5
Amyotrophic lateral sclerosis	11/364	2,11E-05	NUP107 PSMC6 PSMA2 ATP5PB TUBB NEFL NEFM PFN1 NEFH RAB8A PRPH
Dopaminergic synapse	7/132	2,15E-05	GNAO1 GNAL GNG2 GNB1 GNB4 CAMK2G GNAI1
Circadian entrainment	6/97	3,59E-05	GNAO1 GNG2 GNB1 GNB4 CAMK2G GNAI1

Pathways of neurodegeneration	12/475	5,10E-05	PSMC6 PSMA2 ATP5PB TUBB NEFL CTNNB1 NEFM KRAS NEFH CAMK2G RAB8A PRPH
Serotonergic synapse	6/113	8,45E-05	GNAO1 GNG2 GNB1 GNB4 KRAS GNAI1
Glutamatergic synapse	6/114	8,87E-05	GNAO1 GNG2 GNB1 GNB4 SLC17A6 GNAI1
Human cytomegalovirus infection	8/225	9,64E-05	GNAO1 GNA13 GNG2 GNB1 GNB4 CTNNB1 KRAS GNAI1
Glu+IL-4 vs Glu+Vehicle: Upregulated			
Ribosome	13/158	7,93E-09	RPL5 RPL32 RPL12 MRPL19 RPL13A RPL8 RPS4Y1 RPL9 RPS26 RPS25 RPS15A RPS3 RPS13
Coronavirus disease	12/232	4,44E-06	RPS26 RPL5 RPS25 RPS15A RPL32 RPL12 RPS3 RPL13A RPL8 RPS4Y1 RPL9 RPS13
Necroptosis	10/159	5,11E-06	CAMK2B H2AW H2AZ2 CAMK2D PARP1 H2AC20 MACROH2A2 CHMP6 MACROH2A1 SLC25A6
Spliceosome	9/150	2,23E-05	PHF5A SNRPD1 SF3B6 DDX23 DHX15 PLRG1 USP39 LSM3 SNRPB
Salmonella infection	11/249	4,80E-05	VPS18 RALA TUBB2A DYNLT1 EXOC7 KIF5A RPS3 ELMO2 FBXO22 RHOB TXN2
Non-homologous end-joining	3/13	2,69E-04	FEN1 XRCC6 PRKDC
Systemic lupus erythematosus	7/135	4,50E-04	H2AW H2AZ2 SNRPD1 H2AC20 MACROH2A2 MACROH2A1 SNRPB

Glucagon signaling pathway	6/107	7,64E-04	CAMK2B CAMK2D PFKL PKM PFKM PFKP
Ribosome biogenesis in eukaryotes	6/108	8,03E-04	NOP56 FBL NOP58 CSNK2A1 CSNK2A2 GAR1
HIF-1 signaling pathway	6/109	8,42E-04	CAMK2B CAMK2D PFKL ELOB PFKM PFKP
Glu+Minocycline vs Glu+Vehicle: Upregulated			
HIF-1 signaling pathway	2/109	1,87E-03	PFKL CDKN1B
Pentose phosphate pathway	1/30	1,79E-02	PFKL
Galactose metabolism	1/31	1,84E-02	PFKL
Fructose and mannose metabolism	1/33	1,96E-02	PFKL
Fat digestion and absorption	1/43	2,55E-02	MTTP
Arginine and proline metabolism	1/50	2,96E-02	LAP3
Glutathione metabolism	1/57	3,37E-02	LAP3
Glycolysis / Gluconeogenesis	1/67	3,95E-02	PFKL
Central carbon metabolism in cancer	1/70	4,12E-02	PFKL
Chronic myeloid leukemia	1/76	4,47E-02	CDKN1B
Glu+Pioglitazone vs Glu+Vehicle			
Amyotrophic lateral sclerosis	22/364	3,57E-10	RAB1A TUBAL3 TUBB TUBB4B TUBB4A PSMA6 TUBA1B PSMC5 PSMD7 TUBA1A TUBB2A NDUFS8 PSMA2 NDUFS3 SRSF3 NUP54 CYC1 PFN1 HNRNPA1 ACTR10 MCU UBQLN2

Prion disease	19/273	5,76E-10	GSK3B CSNK2A1 TUBAL3 TUBB TUBB4B TUBB4A PSMA6 TUBA1B PSMC5 PSMD7 TUBA1A TUBB2A NDUFS8 PSMA2 NDUFS3 CYC1 SLC25A5 MCU SLC25A6
Parkinson disease	18/249	9,05E-10	TUBAL3 TUBB TUBB4B TUBB4A GNAI1 PSMA6 TUBA1B PSMC5 PSMD7 TUBA1A TUBB2A NDUFS8 PSMA2 NDUFS3 CYC1 SLC25A5 MCU SLC25A6
Alzheimer disease	21/369	2,69E-09	GSK3B CSNK2A1 TUBAL3 TUBB PSEN1 TUBB4B TUBB4A PSMA6 TUBA1B PSMC5 PSMD7 TUBA1A TUBB2A NDUFS8 PSMA2 NDUFS3 CYC1 SLC25A5 GAPDH MCU SLC25A6
Salmonella infection	17/249	6,38E-09	NCKAP1 VPS18 MAP2K4 RAB5B TUBAL3 TUBB TUBB4B TUBB4A RHOB CDC42 TUBA1B TUBA1A TUBB2A RAB9A PFN1 ACTR10 GAPDH
Huntington disease	18/306	2,31E-08	TUBAL3 TUBB TUBB4B AP2A2 TUBB4A PSMA6 TUBA1B PSMC5 PSMD7 TUBA1A TUBB2A NDUFS8 PSMA2 NDUFS3 CYC1 SLC25A5 ACTR10 SLC25A6
Spliceosome	13/150	2,45E-08	ISY1-RAB43 LSM3 LSM2 SART1 RBMXL1 PHF5A DDX39B TRA2B

			SRSF3 DHX16 PPIH HNRNPA1 SNRPA
Pathways of neurodegeneration	22/475	4,63E-08	GSK3B RAB1A CSNK2A1 TUBAL3 TUBB PSEN1 TUBB4B TUBB4A PSMA6 TUBA1B PSMC5 PSMD7 TUBA1A TUBB2A NDUFS8 PSMA2 NDUFS3 CYC1 SLC25A5 ACTR10 MCU SLC25A6
Phagosome	12/152	2,39E-07	RAB5B TUBA1B TUBA1A TUBB2A TUBAL3 TUBB STX7 ATP6V1E1 TUBB4B ATP6V1D TUBB4A VAMP3
mRNA surveillance pathway	9/98	2,25E-06	PNN DAZAP1 DDX19B DDX39B PABPN1 PPP2R1A CPSF3 CSTF2 PAPOLA
